# Pyridine Nucleotide Coenzyme Specificity of *p*-Hydroxybenzoate Hydroxylase and Related Flavoprotein Monooxygenases

**DOI:** 10.3389/fmicb.2018.03050

**Published:** 2018-12-18

**Authors:** Adrie H. Westphal, Dirk Tischler, Florian Heinke, Sarah Hofmann, Janosch A. D. Gröning, Dirk Labudde, Willem J. H. van Berkel

**Affiliations:** ^1^Laboratory of Biochemistry, Wageningen University and Research, Wageningen, Netherlands; ^2^Interdisziplinäres Ökologisches Zentrum, Technische Universität Bergakademie Freiberg, Freiberg, Germany; ^3^Bioinformatics Group Mittweida, University of Applied Sciences Mittweida, Mittweida, Germany

**Keywords:** *Actinobacteria*, coenzyme specificity, fingerprint sequence, flavoprotein, monooxygenase, NAD(P)H, phylogenetic analysis, protein evolution

## Abstract

*p*-Hydroxybenzoate hydroxylase (PHBH; EC 1.14.13.2) is a microbial group A flavoprotein monooxygenase that catalyzes the *ortho*-hydroxylation of 4-hydroxybenzoate to 3,4-dihydroxybenzoate with the stoichiometric consumption of NAD(P)H and oxygen. PHBH and related enzymes lack a canonical NAD(P)H-binding domain and the way they interact with the pyridine nucleotide coenzyme has remained a conundrum. Previously, we identified a surface exposed protein segment of PHBH from *Pseudomonas fluorescens* involved in NADPH binding. Here, we report the first amino acid sequences of NADH-preferring PHBHs and a phylogenetic analysis of putative PHBHs identified in currently available bacterial genomes. It was found that PHBHs group into three clades consisting of NADPH-specific, NAD(P)H-dependent and NADH-preferring enzymes. The latter proteins frequently occur in *Actinobacteria*. To validate the results, we produced several putative PHBHs in *Escherichia coli* and confirmed their predicted coenzyme preferences. Based on phylogeny, protein energy profiling and lifestyle of PHBH harboring bacteria we propose that the pyridine nucleotide coenzyme specificity of PHBH emerged through adaptive evolution and that the NADH-preferring enzymes are the older versions of PHBH. Structural comparison and distance tree analysis of group A flavoprotein monooxygenases indicated that a similar protein segment as being responsible for the pyridine nucleotide coenzyme specificity of PHBH is involved in determining the pyridine nucleotide coenzyme specificity of the other group A members.

## Introduction

*p*-Hydroxybenzoate hydroxylase (PHBH; EC 1.14.13.2) is a group A flavoprotein monooxygenase that catalyzes the *ortho*-hydroxylation of 4-hydroxybenzoate to 3,4-dihydroxybenzoate, a common intermediate step in the degradation of aromatic compounds in soil bacteria (Harwood and Parales, 1996):


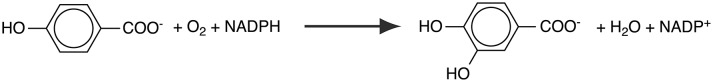


The structural and mechanistic properties of NADPH-specific *Pseudomonas* PHBH have been studied extensively (Entsch and van Berkel, 1995; Entsch et al., 2005; Palfey and McDonald, 2010; Crozier-Reabe and Moran, 2012; Ballou and Entsch, 2013). As a consequence, this enzyme has emerged as the prototype group A flavoprotein hydroxylase (van Berkel et al., 2006; Suemori and Iwakura, 2007; Montersino et al., 2011; Montersino and van Berkel, 2013; Huijbers et al., 2014).

**Figure 1:**
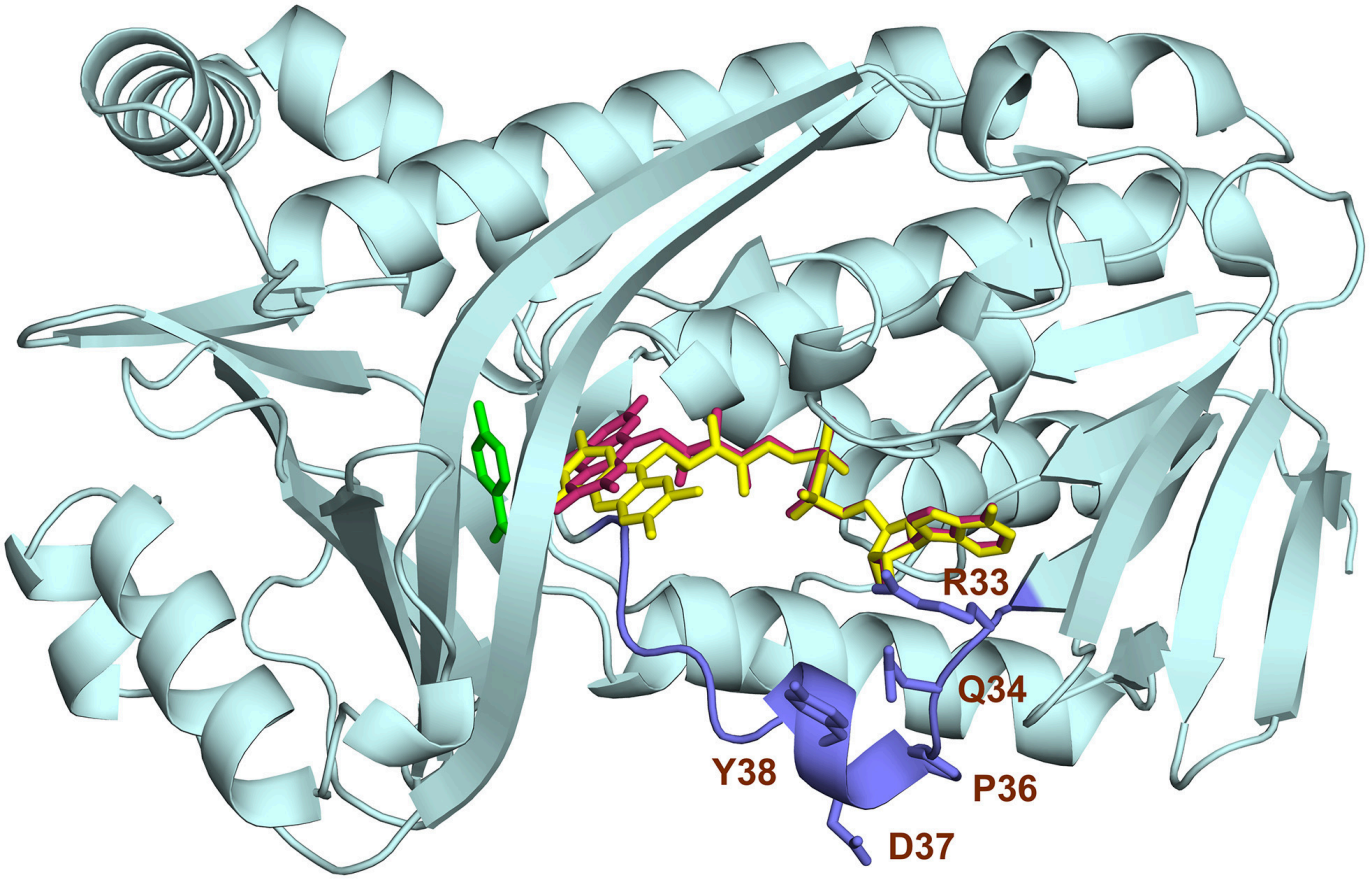
Flavin mobility in *p*-hydroxybenzoate hydroxylase. Cartoon image of the crystal structure of PHBH_*Pf*_ with the FAD cofactor in the *in* (red; pdb entry 1pbe) and *out* (yellow; pdb entry 1pdh) conformation. The substrate is colored green and the region containing helix H2 is colored blue. The indicated amino acids in the helix H2 region have been replaced by site-directed mutagenesis to alter the pyridine nucleotide coenzyme specificity (Eppink et al., 1999).

The isoalloxazine moiety of the flavin cofactor of PHBH is mobile and adopts different positions *in* and *out* the active site (Gatti et al., 1994; Schreuder et al., 1994; Figure 1). Reduction of the flavin by NADPH is assumed to take place in the *out* position (van Berkel et al., 1994; Wang et al., 2002; Ballou and Entsch, 2013). After NADP^+^ release, the reduced flavin moves to the *in* position, where the reaction with oxygen and subsequent hydroxylation of the aromatic substrate occurs. A similar mobility of the flavin cofactor has been observed in other group A flavoprotein monooxygenases, including among others phenol hydroxylase (Enroth et al., 1998), 3-hydroxybenzoate 4-monooxygenase (Hiromoto et al., 2006), and 2-hydroxybiphenyl monooxygenase (Kanteev et al., 2015).

Despite their important biological role (Huijbers et al., 2014), relatively little is known about the occurrence of NADH-preferring PHBHs and how PHBH and its relatives interact with the pyridine nucleotide coenzyme. Unlike many other NAD(P)H-dependent oxidoreductases (Scrutton et al., 1990; Ojha et al., 2007; Cahn et al., 2016, 2017; Sellés Vidal et al., 2018), group A flavoprotein monooxygenases lack a canonical pyridine dinucleotide binding domain (van Berkel et al., 2006; Treiber and Schulz, 2008; Huijbers et al., 2014; Mascotti et al., 2016). For PHBH from *Pseudomonas fluorescens* (PHBH_*Pf*_), an interdomain binding for NADPH was proposed (Eppink et al., 1998a). Based on this binding mode, a switch in coenzyme specificity was achieved by replacing five amino acid residues of the solvent accessible helix H2 of the FAD domain (Figure 1) (Eppink et al., 1999). Support for the interdomain binding of the pyridine nucleotide was obtained from the crystal structure of the R220Q variant of *P. aeruginosa* PHBH in complex with NADPH (Wang et al., 2002). However, this substrate-free complex presented an inactive conformation, which pointed to significant ligand dynamics during the reductive half reaction (Ortiz-Maldonado et al., 2003; Entsch et al., 2005; Westphal et al., 2006; Ballou and Entsch, 2013).

To learn more about the evolutionary relationship of the pyridine nucleotide coenzyme specificity of PHBHs, we here performed a phylogenetic analysis of putative PHBHs and investigated the sequence-function relationship of actinobacterial and proteobacterial PHBHs. The results were used to predict the structural features that determine the pyridine nucleotide coenzyme specificity of other group A flavoprotein monooxygenases.

## Materials and Methods

### Cloning and Sequencing of *Rhodococcus* PHBH Genes

Cultivation of *Rhodococcus opacus* 557 and *Rhodococcus rhodnii* 135 was performed with 4-hydroxybenzoate as sole source of carbon and energy (Jadan et al., 2001). Genomic DNA from *R. opacus* 557 and *R. rhodnii* 135 was prepared from cells obtained after centrifugation of 50 mL cultures, which were subsequently washed with 50 mM Tris-HCl, pH 7.6 and treated with phenol-chloroform to extract the DNA (Sambrook and Russel, 2001). *Escherichia coli* DH5α (GIBCO BRL) and clones obtained were grown while shaking at 37°C in lysogeny broth (LB) medium (Sambrook and Russel, 2001) containing ampicillin (100 μg per mL).

Oligonucleotides were designed and synthesized according to the *N*-terminal and internal sequences of PHBH_*Ro*_ and PHBH_*Rr*_ (Montersino and van Berkel, 2013). In addition, primers were designed using the sequences of conserved regions of PHBH_*Pf*_ (Weijer et al., 1982), and PHBHs from *Acinetobacter* sp. ADP1 (DiMarco et al., 1993) and *Azotobacter chroococcum* (Quinn et al., 2001).

The constructs pROPOB1 and pRRPOB1 were obtained by cloning the 870 bp PCR products of primers fw-Rh557 [GAA (CT)AC CCA (AG)GT (CG)GG CAT (ACT)GT] and rev-pobA [CGGT(GC)G G(GC)G G(GC)A C(AGT)A T(AG)T G] with *R. opacus* 557 or *R. rhodnii* 135 DNA into the *Eco*RV site of pBS T-tailed as described elsewhere [pBluescript II SK(+), Stratagene; (Marchuk et al., 1991)]. Inserts obtained from *Eco*RV digested plasmid DNA were labeled with digoxigenin by using the DIG DNA Labeling and Detection Kit Nonradioactive (Boehringer, Germany) for the detection of fragments on Southern blots of *Eco*RI-digested *R. opacus* 557 or *R. rhodnii* 135 DNA. Respective DNA-fragments were purified from agarose gels, ligated into *Eco*RI-digested and dephosphorylated pBS. The resulting plasmid was transformed into *E. coli* DH5α and obtained colonies checked by colony hybridization as described elsewhere (Eulberg et al., 1997). Positive clones p*Ro*POB1-1 contained a 9.8 kb *Eco*RI fragment of *R. opacus* 557 DNA and p*Rr*POB1-1 a 7.8 kb *Eco*RI fragment of *R. rhodnii* 135 DNA, respectively, comprising the complete *pobA* genes. Subclones containing less flanking DNA regions were obtained by using various restriction endonucleases as shown in Figure S1.

DNA sequencing and sequence analysis was performed with common primers such as T3, T7, M13, or rM13 and respective software as described previously (Gish and States, 1993; Thiel et al., 2005; Felsenstein, 2009).

*Rhodococcus opacus* 1CP is a model strain for the degradation of aromatic compounds (Eulberg et al., 1997) and encodes a single PHBH-like protein (accession number ANS30736) which is 99% similar to the other *Rhodococcus* PHBHs reported herein. The corresponding gene *pobA* was amplified by PCR and cloned into pET16bp as described earlier Riedel et al., 2015. Using the primers *pobA*-fw (5′-catatgaacacacaggtcgggatc-3′) and *pobA*-rev (5′-ggtacctcagcccagcggggtgc-3′) allowed introducing NdeI/NotI restriction sites for cloning. The subsequent cultivation and expression was done as described below for the *Cupriavidus* enzymes.

### Cloning and Expression of *Cupriavidus necator* PHBH Genes

*Ralstonia eutropha* (also designated as *Cupriavidus necator*) JMP134 harbors a number of enzymes involved in degradation or aromatic compounds and amongst those two PHBH-like proteins (accession numbers KX345395 and KX345396 for PHBH_Cn1_ and PHBH_Cn2_, respectively; Pérez-Pantoja et al., 2008. The PHBH-encoding genes AOR50758 and AOR50759 were codon optimized according to the codon table of *Acinetobacter* sp. ADP1, synthetically produced, obtained in a pEX-cloning vector and cloned into pET16pb by methods reported earlier Oelschlägel et al., 2015; Riedel et al., 2015. Cloning was performed using *E. coli* DH5α and LB medium (10 g tryptone, 5 g yeast extract and 10 g NaCl per L) was used with ampicillin (100 μg per ml). For gene expression, the pET construct was transferred to *E. coli* BL21 (DE3) pLysS and cultivated in LB medium containing ampicillin (100 μg per ml) and chloramphenicol (34 μg per ml). Fernbach flasks (1 L) were used and the cultures were grown at 37°C until an OD600 of 0.2 and subsequently cooled to 20°C. At an OD600 of about 0.5 the gene expression was induced by adding IPTG (0.5 mM) and the cultures were continued at 20°C for 20 h. Afterwards cells were harvested by centrifugation (1 h at 5,000 x g, 4°C) and the pellets were stored at −20°C.

### Purification of PHBH Enzymes

The cell pellets were resuspended in 50 mM Tris/sulfate buffer (pH 7.5) while adding 8 units DNaseI (AppliChem—BioChemica, Darmstadt). Cells were broken through ultrasonic treatment (15 times 30 s, 70% power using a HD 2070, MS 72, Bandelin Sonoplus) in an ice-bath. Cell debris was removed by centrifugation (20,000 × g for 20 min, 4°C). After filtration through a cellulose membrane (0.2 μm pore size) to remove remaining particles, the crude extracts were subjected to Ni-chelate chromatography using a 1 ml HisTrap FF column (GE Healthcare) mounted in an ÄKTA fast-performance liquid chromatographer (GE Healthcare). The column was pre-equilibrated with 10 mM Tris/sulfate buffer (pH 7.5). After applying the cell extract, the column was washed with 3 ml loading buffer and then with loading buffer containing 25 mM imidazole until no protein eluted anymore (about 6 ml). Next we started a gradient to achieve 500 mM imidazole in the loading buffer within 6 ml. Target protein eluted during this gradient. Fractions were collected in 1 ml size and checked for standard PHBH activity (see section Enzyme Activity Measurements and Product Analysis). Active fractions were pooled and concentrated and buffer exchanged using Amicon Ultra-15 centrifugal filter devices (30 kDa) in 50 mM Tris/sulfate buffer (pH 7.5) containing 45% glycerol. The enzyme samples were stored at −20°C until further use. Protein concentration was determined by means of a Bradford assay.

### Enzyme Activity Measurements and Product Analysis

Enzyme activity measurements were performed at 30°C in 50 mM Tris/sulfate buffer (pH 7.5), containing 60 μM FAD, 175 μM NAD(P)H (or 0 to 175 μM if varied) and 500 μM 4-hydroxybenzoate (or 0–500 μM if varied). Reactions were started by adding 20–40 nM of enzyme solution. All assays were performed in triplicate and either followed by the decrease in absorption at 340 nm (ε_340_ = 6.22 mM^−1^ cm^−1^) or by HPLC analysis of 3,4-dihydroxybenzoate. For HPLC analysis, five samples were taken at 1 min intervals and reactions were stopped adding ice-cold methanol. Before analysis, samples were centrifuged at 17,000 x g for 2 min to remove protein precipitates. HPLC (10 μl sample volume) was performed with a C18 reverse phase column (Knauer) running in a Ultimate3000 (ThermoScientific) UHPLC system. Elution was done isocratically with 0.1% trifluoroacetic acid, containing 30% methanol (flow rate 1 ml per min; 6 min total run time). Authentic standards of 4-hydroxybenzoate, NAD(P)H, NAD(P)^+^ and 3,4-dihydroxybenzoate were used to calibrate the system. Absorption was continuously monitored at 215 nm and spectra of eluting compounds were acquired with a diode array detector.

### Phylogenetic Analysis

PHBH protein sequence analyses were performed using the NCBI BlastP-service (Altschul et al., 1990). In total, 70 PHBHs of various bacterial phyla were selected and used for *in silico* analyses. The protein sequences from *P. putida* KT2440 (NP_746074; salicylate hydroxylase), *C. testosterone* TA441 (BAA82878; 3-(3-hydroxyphenyl)propionate hydroxylase), *S. chlorophenolicum* L-1 (AAF15368; pentachlorophenol monooxygenase), and *Acinetobacter* sp. ADP1 (AAF04312; salicylate hydroxylase) served as appropriate out-group, as reported earlier (Suemori et al., 2001; Pérez-Pantoja et al., 2008).

The sequence information was used for a phylogenetic analysis allowing functional annotation of PHBH genes. Several algorithms (Fitch-Margoliash, maximum parsimony, maximum likelihood, and neighbor joining) were applied to obtain reliable sequence alignments and representative distance trees. The following software tools were used: Clustal-X (ver. 1.8) (Higgins and Sharp, 1988; Thompson et al., 1997), GeneDoc (ver. 2.6.003), the PHYLIP 3.66 package (PROTDIST and FITCH) (Felsenstein, 2005), and MEGA5 (Tamura et al., 2011). Bootstraps of 1,000 replicates were calculated from the corresponding alignment by means of the PHYLIP 3.66 package (SEQBOOT, PROTDIST, FITCH, and CONSENSE) (Felsenstein, 2005).

Sequence logos were constructed as follows: the PHBH_*Pf*_ protein sequence was used as input query for a BlastP (NCBI) (Altschul et al., 1990) search using the non-redundant protein sequences database. Only sequences with an E-value smaller than 1e^−100^ were selected. After filtering the output sequences for duplicates, crystal structure sequences and cloned protein variants using Sequence Dereplicator and Database Curator (SDDC, ver. 2.0) (Ibrahim et al., 2017), the sequences of the protein segment involved in pyridine nucleotide coenzyme binding were selected and aligned using Clustal Omega (Sievers et al., 2011). The top 200 protein segment sequences were used to generate a sequence logo using the WebLogo server (ver. 2.8.2, Crooks et al., 2004). This process was repeated with the PHBH_*Ro*_ protein sequence as query input.

### Protein Energy Profiling

The phylogenetic analysis and its outcome is of major relevance for the identification of the pyridine nucleotide coenzyme binding sites. The above described methods were validated by the herein described protein energy profiling, which allows for drawing sequence—structure relations (Heinke et al., 2015).

Obtaining energy profiles from protein structures is realized by means of a coarse-grained residue-level pair potential function. Based on the theoretical assumptions elucidated in Wertz and Scheraga (1978), Eisenberg and McLachlan (1986), and Dressel et al. (2007), this energy model approximates the hydrophobic effect by utilizing buried and exposed preferences for each of the 20 canonical amino acids. Given a set of globular protein structures, one can determine the frequencies for each amino acid of being exposed on the outside or buried inside the protein by using the DSSP program (Kabsch and Sander, 1983) as proposed by Ofran and Rost (2003) or by determining residue orientation and local spatial residue packing (Dressel et al., 2007; Heinke and Labudde, 2012). The energy potential (*E*_*i*_) is calculated using the following equations:

(1) $$\begin{array}{rcl} e_{i} & = & -\ln\left(\frac{f_{bur,i}}{f_{exp,i}}\right), \end{array}$$

(2) $$\begin{array}{rcl} e_{ij} & = & e_{i}+e_{j}, \end{array}$$

(3) $$\begin{array}{rcl} E_{i} & = & \sum_{j\in Protein,j\neq i}g\left(i,j\right)\left[e_{ij}\right]. \end{array}$$

Given a residue at sequence index *i*, the single-residue potential *e*_*i*_ is computed using the amino acid-specific buried-exposed frequency ratio (Equation 1). As shown in Equation (2), the pair potential *e*_*ij*_ between two residues at indices *i* and *j* corresponds to the sum of single-residue potentials in this model. Finally, by iterating over all residues that are in contact with residue *i*, the potential *E*_*i*_ is derived (Equation 3). A contact between two residues (*i* and *j*) is assumed, if the Cβ - Cβ atom distance is < 8 Å (in case of Gly, Cα atom coordinates are used as spatial reference points instead).

The sequence of residue energy potentials (*E*_1_,…,*E*_i_,…,*E*_n_) corresponds to the protein's energy profile (Dressel et al., 2007; Heinke and Labudde, 2012, 2013; Heinke et al., 2015). In addition, an algorithm for aligning two energy profiles has been adapted from Mrozek et al. (2007) which, besides detecting similarities and differences of residue energy potentials, can also give a distance scoring function (referred to as dScore) as a measure of global energy profile similarity of two energy profiles *P*_1_ and *P*_2_ (Heinke and Labudde, 2013; Heinke et al., 2015):

(4) $$\begin{array}{r} \text{dScore}\left(P_{1},P_{2}\right)=-\log\left(\frac{x_{r}-{\bar{x}}_{P}}{x_{Opt}-{\bar{x}}_{P}}\right), \end{array}$$

where

(5) $$\begin{array}{r} x_{\text{Opt}}=\frac{\delta \left(\left|{\text{P}}_{1}|+|{\text{P}}_{2}\right|\right)}{2}. \end{array}$$

The dScore corresponds to the normalized energy profile alignment raw score *x*_*r*_with respect to the average score *x*_*P*_ obtained from random energy profiles and the highest possible dScore *x*_*opt*_ of two profiles with lengths |P_1_| and |P_2_|. Here, δ acts as an alignment parameter with δ > 0. The negative logarithm leads to a distance-like formulation, with two identical energy profiles yielding a dScore of 0.

Two PHBH structures PDB ID: 1d7l (Ortiz-Maldonado et al., 1999) and PDB ID: 1bgj (Eppink et al., 1998a) were retrieved from the Protein Data Bank (Rose et al., 2011) and used as modeling templates for automated comparative modeling using Modeler (ver. 9.14) (Eswar et al., 2006).

Seventy PHBH sequences (including 15 sequences of biochemically characterized PHBHs and 55 randomly selected PHBH sequences from various bacteria) were used for automated comparative modeling (average sequence identity of ~50%). For each PHBH sequence, five comparative models were generated from which the model with the best corresponding DOPE score (Eswar et al., 2006) was selected for energy profile calculation. In the first step of energy profile analyses, energy profile distance trees were generated. As shown recently (Heinke and Labudde, 2013; Heinke et al., 2015) such distance trees can indicate functional and structural relations and, in case of PHBHs, can support the proposed molecular evolution. To obtain such distance trees, pairwise energy profile alignments were computed as elucidated and, for each energy profile alignment, the corresponding dScore was derived, leading to an energy profile distance matrix. By utilizing the un-weighted pair group method arithmetic mean (Sokal and Michener, 1958) and neighbor joining (Saitou and Nei, 1987) with the derived distance matrix as input, distance trees were generated.

### Evolutionary Rate Calculation

The Rate4Site tool (ver. 2.01) (Mayrose et al., 2004) was used for determining conserved amino acids in PHBH proteins specific for NADPH and NADH, respectively. Multiple sequence alignments were made from selections containing only sequences of pseudomonads and rhodococci, which were used as input to calculate evolutionary rates for all amino acids applying default settings of Rate4site. The obtained values for conservation were scaled to b-factors ranging between 0 and 100. These b-factors were used to color the image of the crystal structure of PHBH_*Pf*_ as example of a NADPH-specific protein. In a similar way, the image of the model structure of PHBH_*Ro*_ as an example of a NADH-preferring protein, was colored. The program Pymol (ver. 1.4) (Schreudinger, 2011) was used to create structure images.

### NADPH Docking in PHBH From *Pseudomonas fluorescens*

The three-dimensional structure of the PHBH_*Pf*_ monomer with the FAD cofactor in the *out* conformation (PDB ID: 1pdh) was used to access the mode of NADPH binding. Docking was performed using HADDOCK (ver. 2.0) (de Vries et al., 2010). The solvated docking was carried out with the recommended parameters of HADDOCK. A distance restraint of 9.0 Å was set between C4N of NADPH and C4a of the flavin cofactor. For rigid-body energy minimization, 2,000 structures were generated, and the 200 lowest energy solutions were used for subsequent semi-flexible simulated annealing and water refinement. Resulting structures were sorted according to intermolecular energy and clustered using a 6.5 Å cut-off criterion. Subsequent cluster analysis was performed within a 2.0 Å cut-off criterion. The structure with the lowest score was selected for generating an image showing the NADPH binding mode of PHBH_*Pf*_.

### Accession Numbers

PHBH sequences determined in this study are available from the GenBank/EMBL/DDBJ nucleotide sequence databases under accession numbers KF234626 for *R. opacus* 557 and KF234627 for *R. rhodnii* 135.

## Results

### Pyridine Nucleotide Coenzyme Specificity of Biochemically Characterized PHBHs

Most biochemically characterized PHBHs with known amino acid sequence are strictly dependent on NADPH (Table 1). However, PHBH from *R. opacus* 557 (PHBH_*Ro*_) and PHBH from *R. rhodnii* 135 (PHBH_*Rr*_) show a clear preference for NADH (Jadan et al., 2001, 2004). This prompted us to determine the amino acid sequences of PHBH_*Ro*_ and PHBH_*Rr*_ (see Methods). Genomic *R. opacus* 557 DNA contained a 1,179-bp open reading frame coding for a PHBH polypeptide of 392 amino acids. The amino acid sequence predicted from the open reading frame corresponded with the experimentally determined *N*-terminal sequence of the protein (MNTQVGIVGGGPAGLM) and with the *N*-terminal sequence (TDHFRQYPFAWFGILAEAPP) of an internal 25 kDa tryptic fragment. Genomic *R. rhodnii* 135 DNA contained a 1,191-bp open reading frame coding for a PHBH polypeptide of 396 amino acids.

**Table 1:** Pyridine nucleotide coenzyme specificity of biochemically characterized *p*-hydroxybenzoate hydroxylases.

**Source**	**Accession number**	**Cofactor preference**	**References**
*Pseudomonas fluorescens*	CAA48483	NADPH	Howell et al., 1972; Weijer et al., 1982; van Berkel et al., 1992
*Pseudomonas putida WCS358*	CAB64666	NADPH	Bertani et al., 2001
*Pseudomonas fluorescens* IFO14160	BAB20910	NADPH	Suemori et al., 1995, 2001
*Pseudomonas aeruginosa* PAO1	NP_248938	NADPH	Entsch et al., 1988; Entsch and Ballou, 1989
*Acinetobacter* sp. ADP1	YP_046383	NADPH	DiMarco et al., 1993; Fernandez et al., 1995
*Pseudomonas fluorescens* ATCC13525	AAA25834	NADPH	Shuman and Dix, 1993
*Rhizobium leguminosarum* B155	AAA73519	NADPH	Wong et al., 1994
*Azotobacter chroococcum* ATCC9043	AAB70835	NADPH	Quinn et al., 2001
*Corynebacterium glutamicum* ATCC 13032	NP_600305	NAD(P)H	Huang et al., 2008
*Pseudomonas* sp. CBS3	CAA52824	NAD(P)H	Seibold et al., 1996
*Rhodococcus opacus* 557	KF234626	NADH	Jadan et al., 2001, this paper
*Rhodococcus rhodnii* 135	KF234627	NADH	Jadan et al., 2001, this paper
*Rhodococcus opacus* 1CP	ANS30736	NADH	This paper
*Cupriavidus necator* JMP134	KX345395^*^	NAD(P)H	This paper
*Cupriavidus necator* JMP134	KX345396^*^	NADPH	This paper

* *The accession numbers of proteins with N-terminal His_10_-tags obtained of codon optimized genes are provided and correspond to original sequences from the JMP134 genome as follows: KX345395 to YP_298206, and KX345396 to YP_299212 (see Figure S3)*.

**Figure 2:**
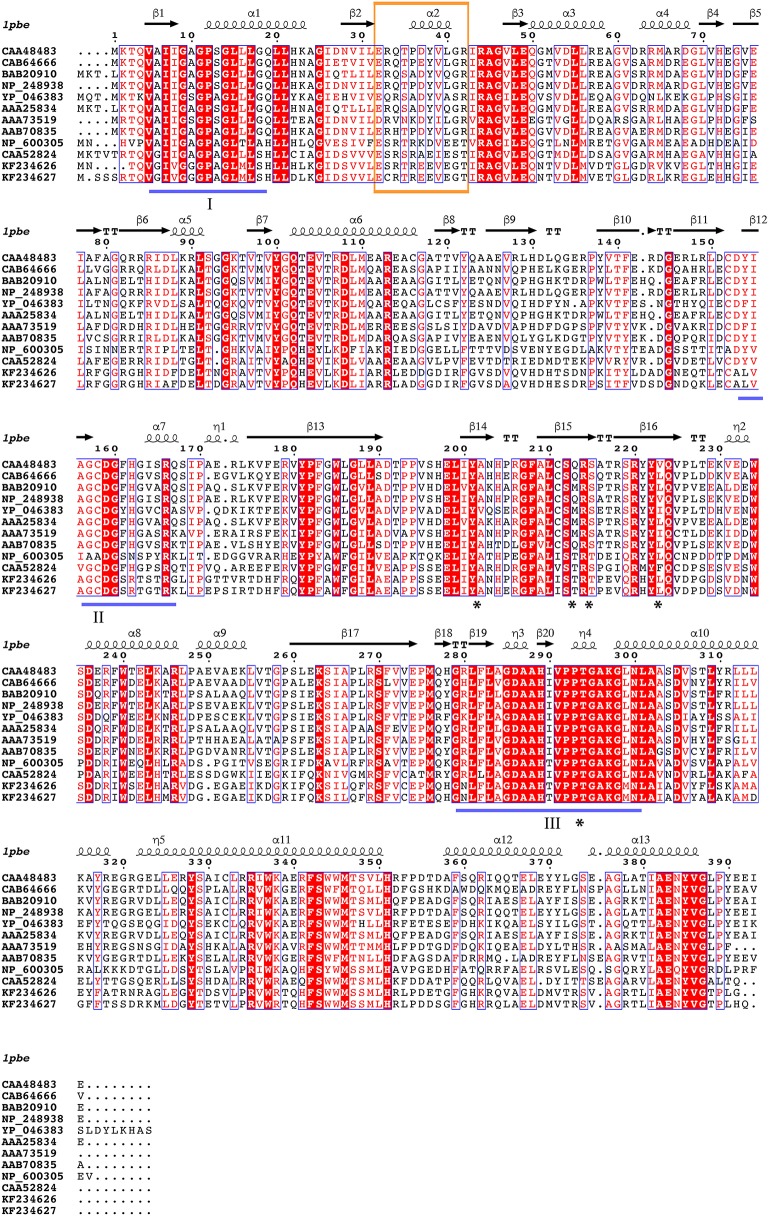
Multiple sequence alignment of selected PHBHs. Accession numbers are given in Table 1. PHBH_*Ro*_ is KF234626 and PHBH_*Rr*_ is KF234627. Identical residues are shown in red. Flavin binding motifs are underlined in blue (I: “GXGXXG”; II: “DG”; III: “GD”; Eppink et al., 1997). Secondary structure assigned from the PHBH_*Pf*_ crystal structure (PDB ID: 1pbe) is indicated above the sequences. The pyridine dinucleotide cofactor recognizing fingerprint region is boxed and residues in direct contact with the substrate are marked with an asterisk. The diagram was produced using ESPript (Robert and Gouet, 2014).

In this paper, amino acid residues are numbered according to the sequence of PHBH_*Pf*_ (CAA48483) to facilitate reference to the 3D-structure. The amino acid sequences of PHBH_*Ro*_ (accession number KF234626) and PHBH_*Rr*_ (accession number KF234627) both share 46.7% identical positions with PHBH_*Pf*_ (Figure 2). Their helix H2 regions, proposed to be involved in determining the pyridine nucleotide coenzyme specificity (Eppink et al., 1999), deviate in amino acid sequence from that of NADPH-specific PHBHs (Figure 2). The latter enzymes typically contain the fingerprint sequence 32-ERxxx(D/E)YVLxR, while the NADH-preferring *Rhodococcus* PHBHs contain the sequence 32-E(S/C)RTREEVEGT.

### Pyridine Nucleotide Coenzyme Specificity of Newly Produced PHBHs

His_10_-tagged forms of two putative PHBHs originating from *C. necator* JMP134 were successfully produced by recombinant expression in *E. coli* BL21 (DE3) and purified by nickel-chelate chromatography (see section Materials and Methods). HPLC experiments confirmed that both isoforms produce 3,4-dihydroxybenzoate as sole product from 4-hydroxybenzoate (Figure S2). Activity measurements with either NADH or NADPH established that PHBH_Cn2_ is strictly dependent on NADPH whereas PHBH_Cn1_ can utilize both coenzymes to perform aromatic hydroxylation. Determination of the apparent kinetic parameters *k*_CAT_ and *K*_M_ (Table 2) through monitoring NAD(P)H consumption as well as 3,4-dihydroxybenzoate production revealed that PHBH_Cn1_ has a slight preference for NADH and that the NADPH-specific PHBH_Cn2_ is about four times more active than PHBH_Cn1_. These experiments also revealed that both enzymes suffer to some extent from uncoupling of substrate hydroxylation resulting in hydrogen peroxide as by-product, thus yielding aromatic product/NAD^+^ ratios of 0.73 and 0.81 for PHBH_Cn1_ and PHBH_Cn2_, respectively.

**Table 2:** Apparent steady-state kinetic parameters of newly produced PHBH enzymes.

**Enzyme**	**Method and corresponding results**					
	**UV/VIS—NAD(P)H consumption**			**HPLC—product formation**		
	***V*_**MAX**_ [U mg^**−1**^]**	***k*_**CAT**_ [s^**−1**^]**	***K*_**M**_ [μM]**	***V*_**MAX**_ [U mg^**−1**^]**	***k*_**CAT**_ [s^**−1**^]**	***K*_**M**_ [μM]**
	**Variable NADH (0–175 μM), constant 4-hydroxybenzoate (500-μM)**					
PHBH_Cn1_	12.3 ± 0.5	9.4 ± 0.4	49.8 ± 5.6	not determined		
PHBH_Ro1*CP*_	20.0 ± 0.9	15.3 ± 0.7	39.7 ± 5.0	not determined		
	**Constant NADH (175 μM), variable 4-hydroxybenzoate (0–500-μM)**					
PHBH_Cn1_	9.9 ± 0.2	7.6 ± 0.2	20.2 ± 2.3	9.0 ± 0.1	6.8 ± 0.1	19.3 ± 1.4
PHBH_Cn2_	**- no activity measurable -**					
PHBH_Ro1*CP*_	16.8 ± 0.3	12.9 ± 0.2	30.4 ± 2.4	21.8 ± 1.0	16.8 ± 0.8	49.9 ± 7.3
	**Variable NADPH (0–175 μM), constant 4-hydroxybenzoate (500-μM)**					
PHBH_Cn2_	49.1 ± 3.7	37.0 ± 2.8	146 ± 20	not determined		
PHBH_Ro1*CP*_	19.8 ± 2.0	15.0 ± 1.5	153 ± 16	not determined		
	**Constant NADPH (175-μM), variable 4-hydroxybenzoate (0–500-μM)**					
PHBH_Cn1_	8.6 ± 0.1	6.5 ± 0.1	19.4 ± 1.2	6.1 ± 0.2	4.6 ± 0.1	20.4 ± 2.5
PHBH_Cn2_	27.0 ± 1.0	18.0 ± 0.7	26.4 ± 4.4	40.0 ± 1.8	30.4 ± 1.5	30.6 ± 5.6
PHBH_Ro1*CP*_	11.3 ± 0.1	8.7 ± 0.1	35.0 ± 1.6	12.5 ± 0.4	9.6 ± 0.3	42.0 ± 4.9

We also determined the pyridine nucleotide coenzyme specificity of the His_10_-tagged form of a putative PHBH from *R. opacus*-1CP (see section Materials and Methods). Kinetic analysis of this enzyme (PHBH_Ro1*CP*_) established a clear preference for NADH (Table 2).

The amino acid sequences of the PHBHs from *C. necator* JMP134 and *R. opacus* 1CP are in agreement with the experimentally determined coenzyme specificities. PHBH_Cn2_ contains the NADPH-preferring sequence motif 32-EQRSPEYVLGR, while PHBH_Ro1*CP*_ contains the NADH-preferring sequence motif 32-ESRTREEVEGT. The NAD(P)H-dependent PHBH_Cn1_ contains the sequence 32-EDCTQAHVEAR.

### PHBH Distribution Among the Tree of Life

Bacteria capable of degrading various aromatic compounds convert the consecutive degradation products into 4-hydroxybenzoate, which then can be funneled into the protocatechuate pathway. Thus, the PHBH enzyme necessary for this route can be expected to be common among microorganisms capable of degrading these aromatic compounds.

Using the amino acid sequence of the NADPH-specific PHBH_*Pf*_ as query sequence for a BlastP search, we identified many putative PHBHs among bacterial phyla with an aerobic lifestyle. Most of them are present in proteobacteria, while roughly 10% is present in *Actinobacteria*. In the other domains of life, PHBH is rarely present. In Archaea, a few putative PHBHs are found, while in Eukarya a small number of hypothetical PHBHs are identified in basidiomycetes such as *Ceratitis capitata*, XP_004528594; *Trichosporon oleaginosus* IBC246, KLT40385; and *Trichosporon asahii var. asahii* CBS 2479, EJT53028. Some of them are similar to PHBH-like proteins of proteobacteria, while others show a high similarity to PHBH-like proteins encoded by *Streptomyces* species (cf. Figure S3a).

**Figure 3:**
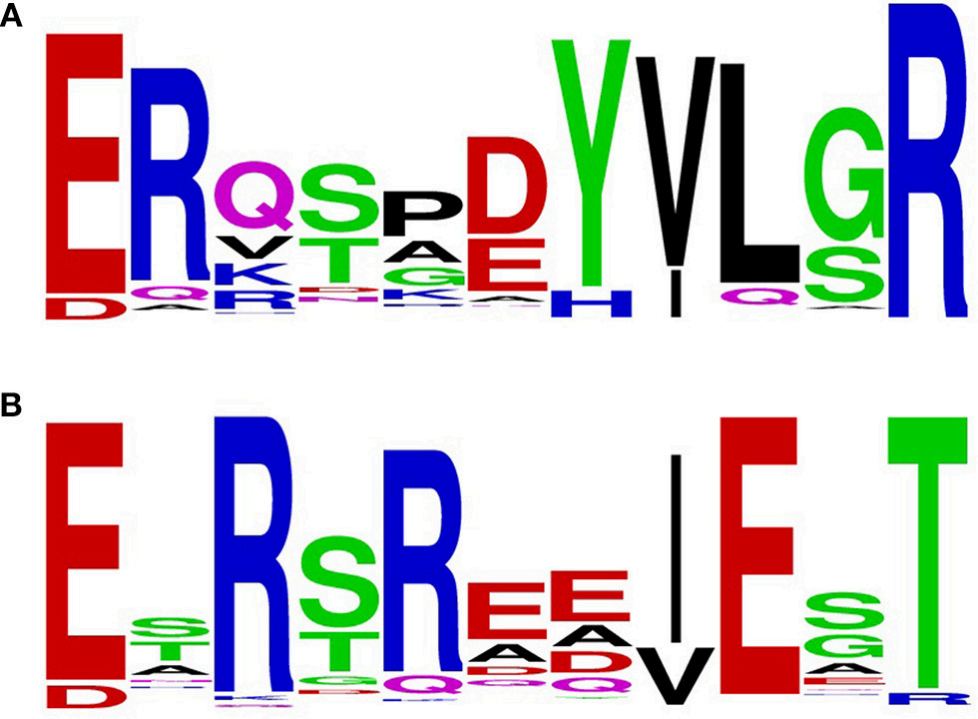
Sequence logos were generated from the aligned sequence parts of PHBHs involved in pyridine nucleotide coenzyme binding using the WebLogo server (ver. 2.8.2, Crooks et al., 2004). **(A)** Sequence logo generated with the NADPH-specific PHBH_*Pf*_ as BlastP query input; **(B)** Sequence logo generated with the NADH-preferring PHBH_*Ro*_ as BlastP query input.

By limiting the BlastP output to an E-value smaller than 1e^−100^, 6135 sequences were retrieved. From these sequences, 1423 had an unique sequence for the loop-helix H2 region. Taking the first 200 sequences of this group for construction of a sequence motif showed that the previously found motif 32-ERxxx(D/E)YVLxR for NADPH specificity is more accurately described by 32-ERx(S/T)x(D/E)YVL(G/S)R (Figure 3A). Similarly, by using the NADH-preferring PHBH_*Ro*_ protein sequence as BlastP query, we found 6,337 sequences with an E-value smaller than 1e^−100^, having 1564 unique loop-helix H2 regions. Taking the first 200 sequences of this group for construction of a sequence motif showed that the NADH-preferring PHBH motif is represented by 32-ExR(S/T)Rxx(I/V)ExT (Figure 3B). After filtering duplicates from the combined total number of 12472 PHBH sequences, 6,482 sequences were unique. Thus, a large overlap exists between the two groups. In the dataset obtained using the PHBH_*Pf*_ sequence, 145 sequences were not present in the dataset obtained with the PHBH_*Ro*_ sequence. Vice versa, 347 sequences were not found in the PHBH_*Pf*_ dataset. The distribution of the sequences not present in each dataset (Figure S3b) shows that most of the sequences only present in the *Ro*-dataset are found among the first 2,000 sequences, while those for the sequences only present in the *Pf*-dataset are located in the last 3,000 sequences of each group.

Interestingly, among the actinobacterial sequences presently available, most comprise the NADH-preferring fingerprint. However, *Mycobacteria* have a mixed type motif, often the first or both arginine(s) of the NADH-fingerprint are present but the remaining part is lacking. In addition, many mycobacterial sequences have parts of the NADPH-preferring fingerprint, especially, x(D/E)YVL(G/S)R. Among the *Streptomyces* sequences many have the NADH-preferring fingerprint, but some also have a mixed type like *Mycobacteria*. However, these mixed-type fingerprints do not have larger parts of the NADPH-fingerprint and are more similar to the sequence of *Cupriavidus* PHBH_Cn1_ and thus might accept both NADH and NADPH. Among rhodococci, the NADH-fingerprint is highly conserved and only a few examples of a mixed type were identified, for example among plant pathogens as *Rhodococcus fascians*, which shows a similar sequence to some *Mycobacteria*. Hereafter, we focused on bacterial PHBHs from which 70 sequences (including the 15 biochemically characterized PHBHs and 55 randomly chosen candidates of various bacteria) were chosen for further analysis of the pyridine nucleotide coenzyme specificity.

### Phylogenetic Analysis

The 70 selected PHBH amino acid sequences and 4 distinct proteins (as out-group as reported elsewhere; Pérez-Pantoja et al., 2008) were used to generate an extended multiple sequence alignment (Figure S1). All sequences in the alignment (except ZP_01743892) harbor the three consensus sequences of flavoprotein hydroxylases involved in FAD binding (Eppink et al., 1997). Furthermore, residues in direct contact with the aromatic substrate are strongly conserved. These residues include Tyr201 and Pro293, which interact with the phenolic moiety, and Ser212, Arg214, and Tyr222, involved in binding the carboxylic group of 4-hydroxybenzoate (Schreuder et al., 1989). With exception of Ser212 (97% Ser, 3% Thr), these residues are 100% conserved.

**Figure 4:**
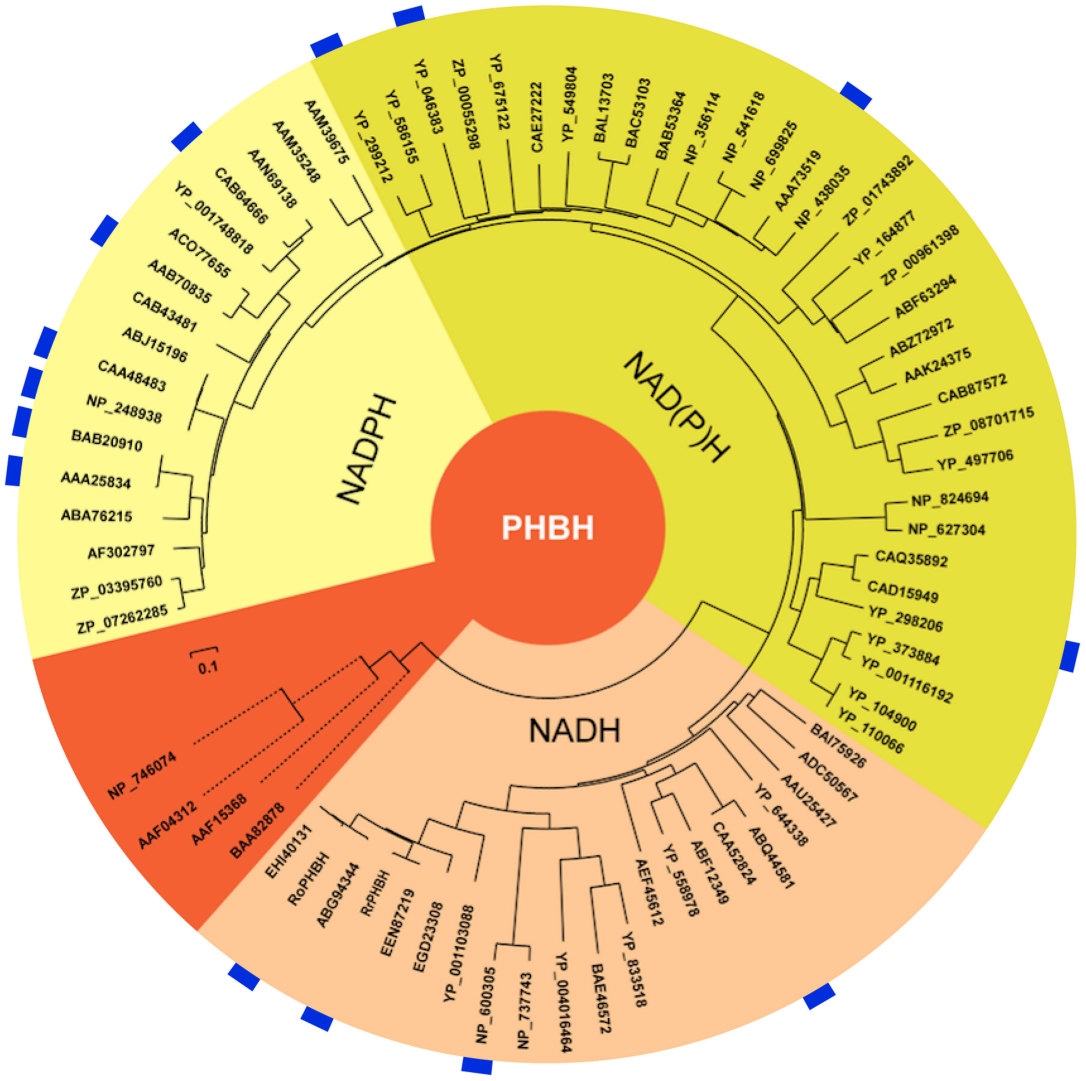
Distance tree illustrating the sequence similarities and predicted pyridine nucleotide coenzyme specificities of PHBHs. The distance tree is based on a similar alignment as that in Figure 2, but now with 74 sequences (Figure S1). The biochemically characterized PHBHs (Table 1) are indicated in blue. The additional accession numbers for (putative) PHBHs are as follows: *Acinetobacter* sp. ADP1, YP_046383; *Agrobacterium tumefaciens* C58, NP_356114; *Arthrobacter* sp. FB24, YP_833518; *Azospirillum* sp. B510, BAI75926; *Azotobacter chroococcum*, AAB70835; *Azotobacter vinelandii* DJ, ACO77655; *Bacillus licheniformis* ATCC 14580, AAU25427; *Bacillus pseudofirmus* OF4, ADC50567; *Bradyrhizobium japonicum* USDA 110, BAC53103; *Bradyrhizobium japonicum* USDA 6, BAL13703; *Brucella melitensis* bv. 1 16M, NP_541618; *Brucella suis* 1330, NP_699825; *Burkholderia mallei* ATCC 23344, YP_104900; *Burkholderia pseudomallei* K96243, YP_110066; *Burkholderia* sp. 383, YP_373884; *Burkholderia vietnamiensis* G4, YP_001116192; *Burkholderia xenovorans* LB400, YP_558978; *Caulobacter crescentus* CB15, AAK24375; *Caulobacter* sp. K31, ABZ72972; *Chelativorans* sp. BNC1, YP_675122; *Citromicrobium* sp. JLT1363, ZP_08701715; *Corynebacterium efficiens* YS-314, NP_737743; *Corynebacterium* sp. ATCC 13032, NP_600305; *Corynebacterium* sp. ATCC 51369, BAE46572; *Cupriavidus metallidurans* CH34, YP_586155; *Cupriavidus metalliredurans* CH34, ABF12349; *Frankia* sp. EuI1c, YP_004016464; *Magnetospirillum* sp. MS-1, ZP_00055298; *Mesorhizobium loti* MAFF303099, BAB53364; *Novosphingobium* sp. DSM 12444, YP_497706; *Polaromonas* sp. JS666, YP_549804; *Pseudomonas aeruginosa* PAO1, NP_248938; *Pseudomonas aeruginosa* UCBPP-PA14, ABJ15196; *Pseudomonas fluorescens* IFO14160, BAB20910; *Pseudomonas fluorescens* Pf0-1, ABA76215; *Pseudomonas fluorescens*, CAA48483; *Pseudomonas putida* KT2440, AAN69138; *Pseudomonas putida* W619, YP_001748818; *Pseudomonas putida* WCS358, CAB64666; *Pseudomonas* sp. ATCC 13525, AAA25834; *Pseudomonas* sp. CBS-3, ABQ44581; *Pseudomonas* sp. CBS3, CAA52824; *Pseudomonas* sp. IMT40, AF302797; *Pseudomonas* sp. strain HR199, CAB43481; *Pseudomonas syringae syringae* 642, ZP_07262285; *Pseudomonas syringae tomato* T1, ZP_03395760; *Ralstonia eutropha* JMP134, YP_298206; *Ralstonia eutropha* JMP134, YP_299212; *Ralstonia solanacearum* GMI1000, CAD15949; *Ralstonia solanacearum* MolK2, CAQ35892; *Rhizobium leguminosarium* B155, AAA73519; *Rhodococcus equi* ATCC 33707, EGD23308; *Rhodococcus erythropolis* SK121, EEN40131; *Rhodococcus jostii*, ABG94344; *Rhodococcus opacus* 557, KF234626; *Rhodococcus opacus* PD630, EHI40131; *Rhodococcus rhodnii* 135, KF234627; *Rhodopseudomonas palustris* CGA009, CAE27222; *Roseovarius nubinhibens* ISM, ZP_00961398; *Rubrobacter xylanophilus* DSM 9941, YP_644338; *Rugeria pomeroyi* DSS-3, YP_164877; *Rugeria* sp. TM1040, ABF63294; *Saccharopolyspora erythraea* sp. NRRL 2338, WP_009944246.1; *Sagittula stellata* E-37, ZP_01743892; *Serratia plymuthica* AS9, AEF45612; *Sinorhizobium meliloti* 1021, NP_438035; *Sphingomonas* sp. LB126, CAB87572; *Streptomyces avermitilis* MA-4680, NP_824694; *Streptomyces coelicolor* A3(2), NP_627304; *Xanthomonas axonopodis citri* 306, AAM35248; *Xanthomonas campestris* ATCC 33913, AAM39675. Some of the PHBH-like hypothetical proteins have been annotated as putative 2-polyprenyl-6-methoxyphenol hydroxylases. The start codons of the sequences of *Rubrobacter xylanophilus* DSM 9941, YP_644338, and *Cupriavidus metallidurans* CH34, YP_586155, were set manually in accordance to the other sequences in the alignment. Sequences from *P. putida* KT2440 (NP_746074; salicylate hydroxylase), *C. testosterone* TA441 [BAA82878; 3-(3-hydroxyphenyl) propionate hydroxylase], *S. chlorophenolicum* L-1 (AAF15368; pentachlorophenol monooxygenase), and *Acinetobacter* sp. ADP1 (AAF04312; salicylate hydroxylase) were used as out-groups (orange).

As already indicated by the pairwise similarity data, the distance tree of bacterial PHBHs (Figure 4) does not reflect the taxonomic relationships, in contrast to what one might expect for a chromosomally encoded enzyme. While some branches in the distance tree represent sequences of only relatively closely related strains, such as various *Burkholderia* strains or various *Rhodococcus* strains, other branches represent relatively closely related PHBHs from taxonomically distant bacteria (e.g., from the phyla of proteobacteria *Acinetobacter* sp. ADP1, *C. necator* JMP134, *Polaromonas* sp. JS666, *Mesorhizobium loti* MAFF303099, and *Rhodospeudomonas palustris* CGA009).

Interestingly, the distance tree clearly reflects the pyridine nucleotide coenzyme preference shown in Table 1. All NADPH-specific PHBHs are located on one side of the tree and on the opposite side the NADH-preferring enzymes are clustered. In between these types we mostly find PHBHs for which a pyridine nucleotide coenzyme preference is not proven yet. However, this preference can be predicted from the phylogenetic tree, and we conclude that representatives closer to the NADH-assigned PHBHs can use both coenzymes, with a preference for NADH. We experimentally confirmed this conclusion by determining the pyridine nucleotide specificity of PHBH_Cn1_, a newly produced representative of this group (Table 2). In the other part of the tree closer to the NADPH-assigned enzymes, PHBHs may also use both pyridine nucleotides but tend to be stricter or even exclusively dependent on NADPH. The out-group of the distance tree includes NAD(P)H-dependent enzymes (Pérez-Pantoja et al., 2008) and intersects the NAD(P)H using putative PHBHs close to the NADH-preferring PHBH type. From an evolutionary point of view this makes sense since a PHBH-predecessor protein might have used both nicotinamide cofactors or even had a preference for NADH. However, more questions on the PHBH evolution need to be answered, e.g., has the pyridine nucleotide coenzyme preference happened by chance or by adaptation, and why does it seem to be stable among certain bacteria, especially among *Actinobacteria*? Most *Actinobacteria* show a NADH-preferring fingerprint or a slightly altered one with the exception of *Mycobacteria*. This might be related to lifestyle and environment of those bacteria, which needs further investigations.

### Energy Potentials of Residues Determining the Pyridine Nucleotide Coenzyme Specificity of PHBH

To get more insight into the evolutionary relationship of the pyridine nucleotide coenzyme specificity, we extracted energy potentials of residues located in the PHBH coenzyme fingerprint motifs from energy profile datasets. Pairwise alignments of these sub-energy profiles have been computed and used for deriving dScores which, similar to the strategies elucidated in the Materials and Methods section, have been processed by un-weighted pair group method arithmetic mean clustering (Figures S4, S5) or neighbor joining hierarchical clustering (Figures S6, S7). A multiple sequence alignment-like representation of these energy potentials (Figures S8, S9) illustrates a strong relationship between residue composition, pyridine nucleotide coenzyme specificity, and energetic properties. First, it becomes clear that conserved residues in these motifs yield a conservation of their energetic state, with most energy potentials being relatively low. It can be proposed that these energetically conserved residues serve as fold stabilizing elements in these motifs as well as in the intra-molecular environment of helix H2. Compared to NADPH-specific and NAD(P)H-dependent PHBHs, NADH-preferring PHBHs yield a high-energetic, unstable environment (Figure S10), which is energetically determined by the presence of two conserved Glu-residues and variable positions which are predominantly occupied by destabilizing residues, such as Asp, Glu and Arg (Zhou and Zhou, 2004). In contrast to these findings, residues in the coenzyme fingerprint motif of NADPH-specific and NAD(P)H-dependent PHBHs yield comparatively low energy potentials and thus are partly stabilizing the binding moiety. It can be concluded that this deviation in molecular stability can contribute to the pyridine nucleotide coenzyme specificity and is an important driver of PHBH evolution.

### Evolutionary Rate of NADPH-Specific and NADH-Preferring PHBHs

We used the Rate4site program (Materials and Methods section) to assess the evolutionary rate of NADPH-specific and NADH-preferring PHBHs. Figure S11 shows that the NADH-enzymes have more regions (colored red) susceptible to mutation compared to the NADPH-enzymes. Indeed, also the loop region with the coenzyme-binding motif is a little more mutation sensitive in the NADH-preferring enzymes, indicative of a strong selection favoring specific amino acids in the NADPH-specific enzymes.

### Pyridine Nucleotide Coenzyme Binding

**Figure 5:**
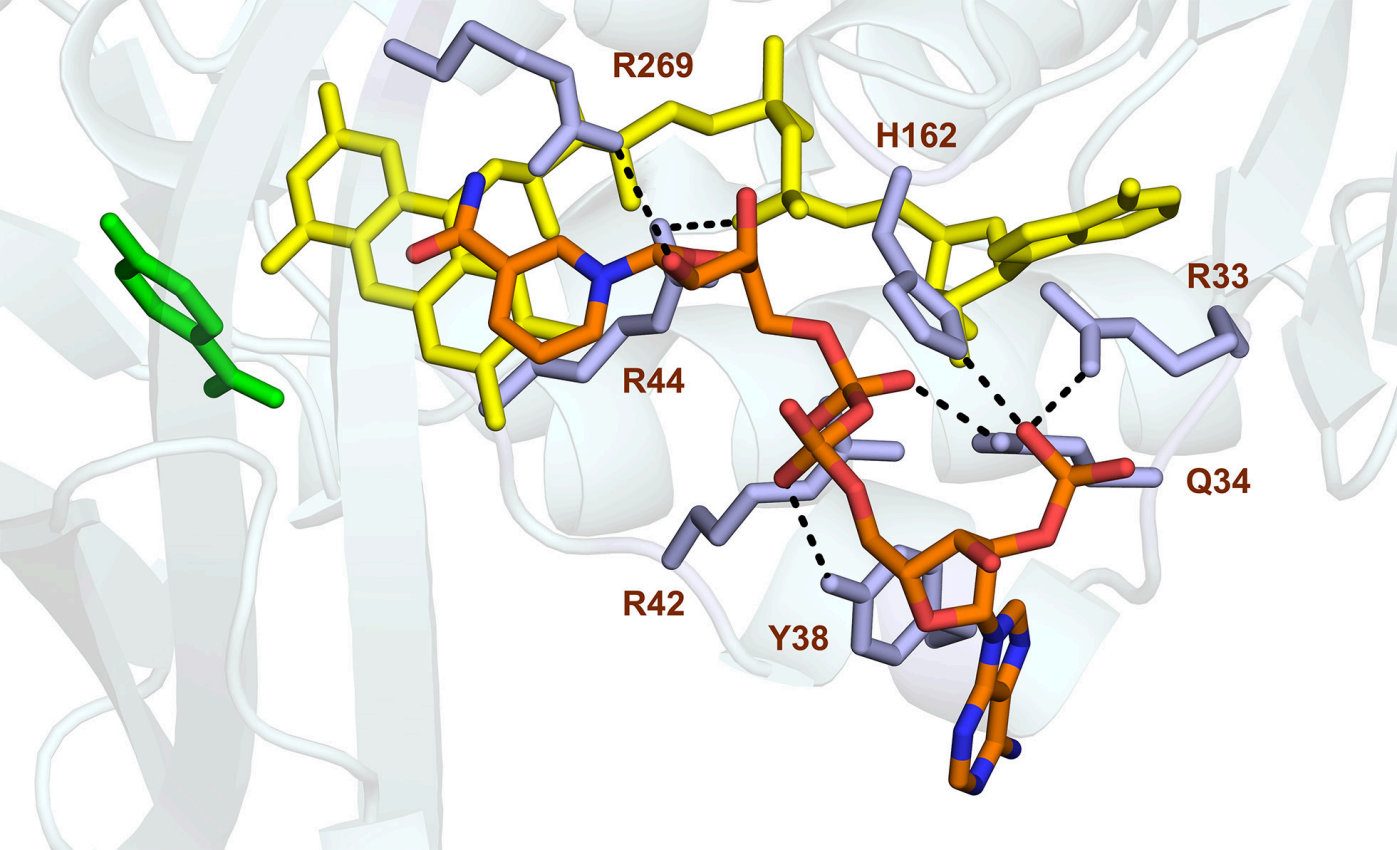
Model of NADPH binding in PHBH_*Pf*_. Cartoon image of the PHBH protein chain in light blue, substrate in green, FAD cofactor (in the *out* position) in yellow and the docked NADPH colored by element. Amino acid residues Arg33, Gln34, Tyr38, Arg42, Arg44, His162, and Arg269, putatively involved in NADPH binding, are shaded in mauve. Hydrogen bonds are indicated by black dashes.

Studies from PHBH variants generated using site-directed mutagenesis support the idea that Tyr38 and Arg42 of helix H2 confer the specificity of PHBH_*Pf*_ for NADPH (Eppink et al., 1998b, 1999; Huang et al., 2008). Based on these findings and the fact that the nicotinamide ring of NADPH binds at the *re*-side of the flavin (Manstein et al., 1986), we docked the NADPH in the enzyme-substrate complex of PHBH_*Pf*_ with the isoalloxazine moiety of the FAD cofactor oriented in the *out* conformation. As can be seen from Figure 5, the docking predicts that His162 and Arg269 interact with the pyrophosphate moiety of NADPH (Eppink et al., 1998a; Wang et al., 2002) and that Arg33, Tyr38 and Arg42 of the NADPH-specific fingerprint sequence 32-ERx(S/T)x(D/E)YVL(G/S)R are involved in orienting the adenosine 2′-phosphate part of NADPH (Eppink et al., 1999).

### Pyridine Nucleotide Coenzyme Specificity in Related Enzymes

**Figure 6:**
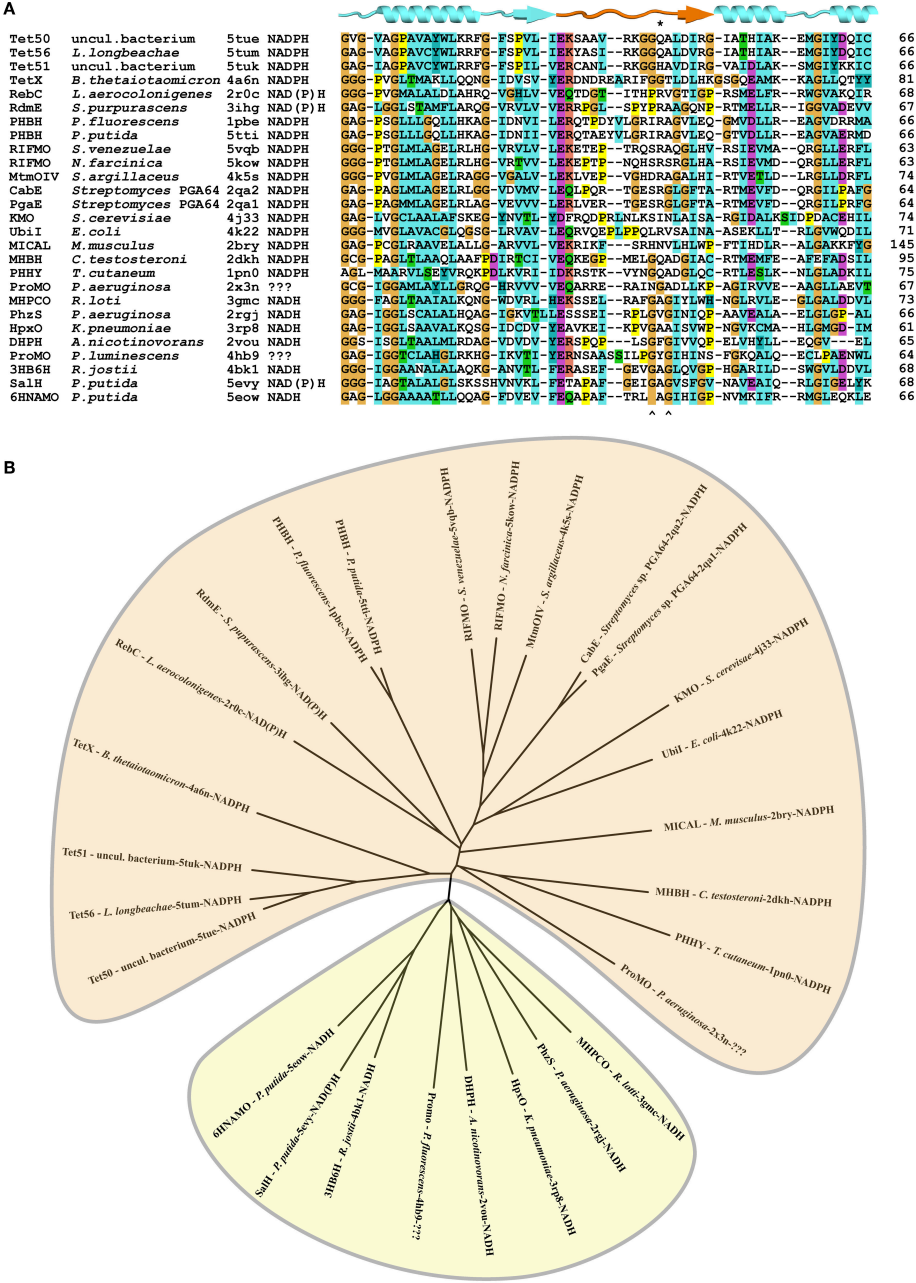
Conservation of loop segment putatively involved in determining the pyridine nucleotide coenzyme specificity of group A flavoprotein monooxygenases. **(A)** Alignment of the sequences forming the loop structures putatively involved in NAD(P)H binding. Alignment of sequences was made using Clustal-X. PDB-entry codes of the sequences used: 5tue, uncultured bacterium, tetracycline destructase (Tet50); 5tum, *Legionella longbeachae*, tetracycline destructase (Tet56); 5tuk, uncultured bacterium, tetracycline destructase (Tet51); 4a6n, *Bacteroides thetaiotaomicron*, tetracycline degrading monooxygenase (TetX); 2r0c, *Lechevalieria aerocolonigenes*, rebeccamycin biosynthetic enzyme (RebC); 3ihg, aklavinone-11-hydroxylase (RdmE); 1pbe, *Pseudomonas fluorescens*, 4-hydroxybenzoate-3-hydroxylase (PHBH); 5tti, *Pseudomonas putida*, 4-hydroxybenzoate-3-hydroxylase (PHBH); 5vqb, *Streptomyces venezuelae*, rifampicin monooxygenase (RIFMO); 5kow, *Nocardia farcinica*, rifampicin monooxygenase (RIFMO); 4k5s, *Streptomyces argillaceus*; Baeyer-Villiger monooxygenase (MtmOIV); 2qa2, *Streptomyces* sp. PGA64, aromatic hydroxylase (CabE); 2qa1, *Streptomyces* sp. PGA64, aromatic hydroxylase (PgaE); 4j31, *Saccharomyces cerevisiae*; kynurenine monooxygenase (KMO);4k22, *Escherichia coli*, 3-octaprenylphenol 2-monooxygenase (UbiI);2bry, *Mus musculus*, catalytic region of molecule interacting with CasL (MICAL);2dkh, *Comamonas testosteroni*, 3-hydroxybenzoate hydroxylase (MHBH); 1pn0, *Trichosporon cutaneum*, phenol hydroxylase (PHHY); 2x3n, *Pseudomonas aeruginosa*, probable FAD-dependent monooxygenase (ProMO); 3gmc, *Mesorhizobium loti*, 2-methyl-3-hydroxypyridine-5-carboxylic acid oxygenase (MHPCO); 2rgj, *Pseudomonas aeruginosa*, phenazine-modifying monooxygenase (PhzS; 3rp8, *Klebsiella pneumoniae* MGH 78578, urate oxidase (HpxO); 2vou, *Arthrobacter nicotinovorans*, 2,6-dihydroxypyridine-3-hydroxylase (DHPH); 4hb9, *Photorhabdus luminescens*, probable FAD dependent monooxygenase (ProMO); 4bk1, *Rhodococcus jostii* RHA1, 3-hydroxybenzoate 6-hydroxylase (3HB6H); 5evy, *Pseudomonas putida*, salicylate hydroxylase (SalH); 5eow, *Pseudomonas putida* KT2440, 6-hydroxynicotinic acid 3-monooxygenase (6HNAMO). Secondary structure elements are indicated above the alignment, with the loop segment colored orange and the amino acid residue number of the last element in the alignment is listed for each sequence. The asterisk (*) indicates the position of R44 of *Pf*-PHBH and the circumflexes (∧) indicate the glycine residues of the GxG motif in other sequences. The secondary structural elements above the sequences refer to the structure shown in Figure 1. **(B)** Distance tree related to the alignment of the sequences forming the loop structures putatively involved in determining the coenzyme specificity of group A flavoprotein monooxygenases. Enzymes in the beige area (top) prefer NADPH, those in the yellowish area (bottom) prefer NADH as coenzyme.

At present, crystal structures of 28 different group A flavoprotein monooxygenases are available in the Protein Data Bank, and for most of these enzymes, the preference for the nicotinamide cofactor is known. Structural alignment of the subfamily members showed similar folds for the FAD and substrate binding domains, which is indicative for a conserved interdomain binding mode of the NAD(P)H coenzyme (Treiber and Schulz, 2008). We aligned the loop segments of these enzymes, putatively involved in NAD(P)H binding, based on the structural position of the adenine moiety of the FAD cofactor and the N- and C-termini of these loops. The alignment obtained from the loop segment sequences (Figure 6A) suggests that the proteins can indeed be grouped in NADPH- or NADH-dependent enzymes and the associated distance tree shows this feature as two separate clusters (Figure 6B). The NAD(P)H-dependent enzymes are located in both the NADH- and NADPH-cluster. Based on type of cluster, the “putative monooxygenase” from *P. luminescens* (PDB ID: 4hb9) is likely NADH-dependent, whereas the “putative monooxygenase” from *P. aeruginosa* (PDB ID: 2x3n) is likely NADPH-dependent.

Within the whole subfamily, there is no clear consensus motif present for NADH- or NADPH-dependency. However, most NADPH-dependent enzymes have an arginine at position 44 (PHBH_*pf*_ numbering), which is capable of H-bond formation, in contrast to the corresponding residue in the NADH-group. The NADH-group has instead a 'GxG' motif near the end of the loop, with x being mostly a hydrophobic residue.

**Figure 7:**
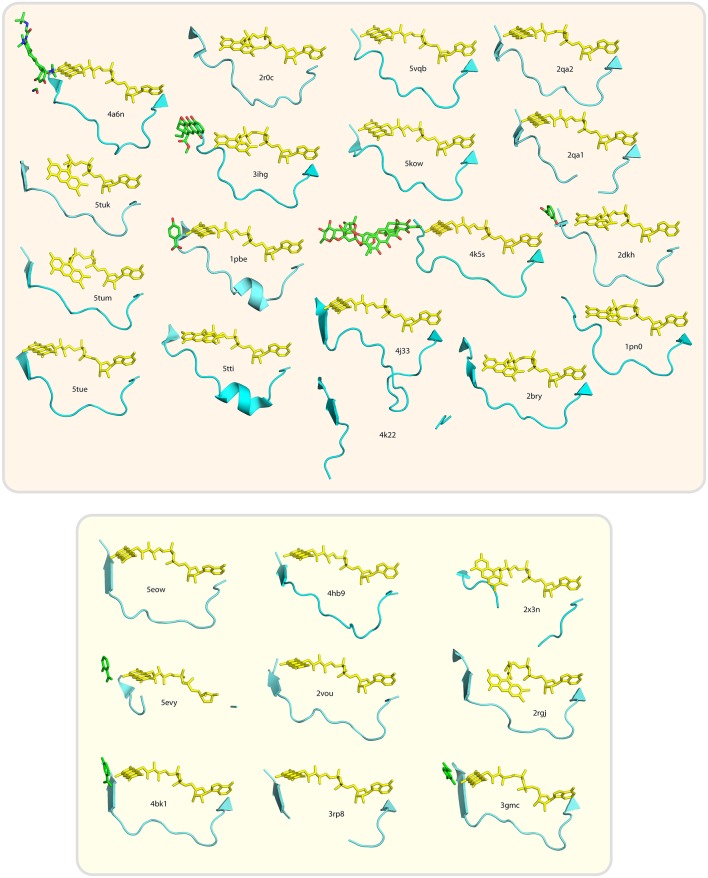
Cartoon images of loop segment structures putatively involved in NAD(P)H binding of group A flavoprotein monooxygenases. Structures in the beige box (top) are NADPH-preferring enzymes, those in the yellowish box (bottom) are NADH-preferring enzymes. Substrates or inhibitors present in the used crystal structures are sketched in green.

The loop segments do not show a clear consensus structure (Figure 7). Those from PHBH enzymes contain a small helix, but none of the others structures have this feature. A few structures are missing some amino acid residues in the loop segment, due to low electron density in the diffraction dataset, which indicates that here the loop is flexible. This flexibility might change upon NAD(P)H binding, which could be essential to allow for the isoalloxazine moiety movement of FAD (i.e., “in/out” conformation).

## Discussion

This paper provides new insights into the pyridine nucleotide coenzyme specificity and evolutionary relationship of PHBH. Based on the known coenzyme preferences of a limited amount of biochemically characterized PHBHs and phylogenetic analysis of putative PHBHs, sequence logos for NADPH-specific and NADH-preferring enzymes could be inferred. The pyridine nucleotide coenzyme specificities of newly produced proteobacterial and actinobacterial PHBHs are in agreement with our phylogenetic analysis, which shows that PHBHs group into three clades comprising sequences of NADPH-specific, NAD(P)H-dependent and NADH-preferring enzymes. The present findings also support that the 2'-phosphate of NADPH does not interact with the side chain of Arg44 (Wang et al., 2002), but binds more close to Tyr38 and Arg42 (Eppink et al., 1999).

Energy profiling established that NADH-preferring PHBHs yield a high-energetic unstable environment around helix H2. This supports that this environment is a predominant site for evolutionary adaptations and leads us to suggest that the pyridine nucleotide coenzyme specificity linked to this sequence has evolved differently according to the evolutionary pressure in the host cell.

It has been estimated that the FAD-binding domain of flavoprotein monooxygenases appeared in coincidence with the emergence of aerobic metabolism, around 2.9 billion years ago (Mascotti et al., 2016). Because both nicotinamide cofactors were already present, the pyridine nucleotide coenzyme specificity of PHBH must have evolved later. What can we learn from the present study regarding the evolutionary history of the pyridine nucleotide coenzyme specificity of PHBH?

First, we raised the question on convergent or divergent evolution. Especially, since NADH is mainly involved in catabolic and NADPH in anabolic pathways, one might argue that two different ancestor proteins arose from different pathways, which led by convergent evolution to PHBH-like proteins but with different nicotinamide cofactor dependency. However, the extensive phylogenetic analysis and alignments made herein do not support this theory since all (putative) PHBHs have highly similar sequences, a comparable length, conserved secondary structure elements and thus a similar fold. Therefore, a divergent evolution of PHBHs from one predecessor must have led to the differences in nicotinamide cofactor dependency. The phylogenetic distance tree suggests that the PHBH ancestor could use both nicotinamide co-substrates and the NADH-preferring PHBHs are supposed to be closer related to this predecessor and therewith the older enzymes (Figure 4). Thus NADPH-specific PHBHs have likely evolved more recently.

Next, we asked ourselves if this evolutionary event occurred by chance or by adaptation (Zhu et al., 2005). As noted above, most of the NADH-preferring (putative) PHBH enzymes are harbored by *k*-strategists as actinobacterial *Rhodococcus, Corynebacterium*, or *Streptomyces* species (Juteau et al., 1999; Margesin et al., 2003; Singer et al., 2011). These microorganisms can handle nutrient limited and highly populated environments, known to be stress tolerant, have a huge catabolic power, and are slow in reproducing. On the other hand, *r*-strategists such as proteobacterial *Pseudomonas* and *Acinetobacter* species (Margesin et al., 2003), reproduce fast, colonize quickly nutrient rich environments, form less stable populations and are attractive prey for other organisms. They need to adapt to a certain environment very fast; thus, they can reproduce in a sufficient manner to ensure survival of their species. Interestingly, all NADPH-specific PHBH proteins are harbored by those *r*-strategists. Moreover, some of these pseudomonads are known to need high levels of NADPH for generating a reductive environment (Singh et al., 2007, 2008). The prevalence of NADPH in such organisms could have caused an adaptive, stepwise evolution toward NADPH-dependence of PHBH enzymes. This is also in agreement with the fact that mutations of few amino acids already change the nicotinamide cofactor preference (Eppink et al., 1999).

Based on phylogeny and lifestyle of various PHBH harboring bacteria we propose that the pyridine nucleotide coenzyme specificity of PHBH has emerged through adaptive evolution. It can be assumed that the PHBH ancestor could use both nicotinamide cofactors with a preference for NADH as source of reducing equivalents. In rhodococci, which are *k*-strategists characterized by slow doubling times (Kurosawa et al., 2010) and in general a high stress tolerance, the NADH-dependent PHBHs retained. These enzymes are the older versions of PHBH. In case of *r*-strategists, which possess a high energy-consuming lifestyle, the available NADPH acted likely as a driving force to evolve strictly NADPH-dependent PHBHs. These enzymes are supposed to have evolved more recently. Thus, we can state that NADPH converting PHBHs have evolved by adaptation to their host and therewith present the youngest PHBH enzymes.

Our data indicate that group A flavoprotein monooxygenases all share with PHBH a similar mode of NAD(P)H binding. However, the here identified pyridine nucleotide coenzyme recognition motifs are specific for PHBH enzymes. Other group A flavoprotein monooxygenases (Huijbers et al., 2014); (Mascotti et al., 2016) likely contain similar motifs, but the sparse availability of biochemical data on the pyridine nucleotide coenzyme specificity of these enzymes does not allow for a reliable prediction of these motifs.

## Conclusion

In this paper, we have described new insights into the pyridine nucleotide coenzyme specificity of *p*-hydroxybenzoate hydroxylase (PHBH) and related group A flavoprotein monooxygenases. By integrating data from phylogeny, structural modeling and enzyme kinetics, it was established that PHBHs group into three clades consisting of NADPH-specific, NAD(P)H-dependent and NADH-preferring enzymes. Furthermore, the results suggest that the NADPH-specific enzymes evolved through an adaptive process from NADH-preferring enzymes and that the loop segment responsible for the pyridine nucleotide coenzyme specificity of PHBH is also involved in the pyridine nucleotide coenzyme specificity of the other group A members. The present work might stimulate future studies directed at understanding the pyridine nucleotide coenzyme specificity of group A flavoprotein monooxygenases in molecular detail.

## Author Contributions

AW and DT carried out the phylogenetic analysis. AW performed the structural alignments and docking experiments. FH and DL carried out the energy potential profiling and evolutionary rate analysis. The cloning and expression of *pob* genes was performed by SH and JG. Purification and biochemical characterization of enzymes was done by AW and SH. AW, DT, FH, and WB wrote the manuscript. All authors read and approved the final manuscript.

### Conflict of Interest Statement

The authors declare that the research was conducted in the absence of any commercial or financial relationships that could be construed as a potential conflict of interest.

## Abbreviations

PHBH: *p*-hydroxybenzoate hydroxylase
PHBH_*Pf*_: PHBH from *Pseudomonas fluorescens*
PHBH_*Ro*_: PHBH from *Rhodococcus opacus* 557
PHBH_*Rr*_: PHBH from *Rhodococcus rhodnii* 135
PHBH_Ro1*CP*_: PHBH from *Rhodococcus opacus* 1CP
PHBH_Cn1_: PHBH-1 from *Cupriavidus necator* JMP134
PHBH_Cn2_: PHBH-2 from *Cupriavidus necator* JMP134.
