# Supplementary material for: Pyridine Nucleotide Coenzyme Specificity of p-Hydroxybenzoate Hydroxylase and Related Flavoprotein Monooxygenases
Source: Front Microbiol. 2018 Dec 18;9:3050. doi: 10.3389/fmicb.2018.03050 (PMC6315137; doi:10.3389/fmicb.2018.03050)
Supplement: Supplementary file 1 [file Data_Sheet_1.pdf]

Supplementary Material

Coenzyme specificity of *p*-hydroxybenzoate hydroxylase and related flavoprotein monooxygenases

Adrie Westphal[1], Dirk Tischler[2,3], Florian Heinke[4], Sarah Hofmann[2], Janosch A. D. Gröning[2,5], Dirk Labudde[4], Willem J. H. van Berkel[1]

[1]Laboratory of Biochemistry, Wageningen University & Research, Stippeneng 4, 6708 WE Wageningen, The Netherlands

[2]Interdisziplinäres Ökologisches Zentrum, Technische Universität Bergakademie Freiberg, Leipziger Str. 29, 09599 Freiberg, Germany

[3]Present address: Microbial Biotechnology, Ruhr University Bochum, Universitätsstr. 150, 44780 Bochum, Germany

[4]Bioinformatics Group Mittweida, University of Applied Sciences Mittweida, Technikumplatz 17, D-09648 Mittweida, Germany

[5]Present address: Institut für Mikrobiologie der Universität Stuttgart, Allmandring 31, D-70569 Stuttgart, Germany

Contents:

Protein energy profiling

Supplementary figures S1-S11

References

1 Protein energy profiling

To evaluate the phylogenetic analysis described in the main manuscript, we conducted a complementary approach including structural modelling and energy profiling of obtained 3D-models. The latter was then applied for additional phylogenetic analysis (see also Materials and Methods section) as well as identifying structural conserved motifs.

Energy profile distance trees generated by the unweighted pair group method with arithmetic mean (UPGMA; Fig. S2 and Fig. S3) support the functional classification of PHBHs as proposed from the phylogenetic analysis (see Fig. 4 main manuscript). Most notably, the UPGMA distance tree generated from structures modelled with 1bgj as template (Fig. S3) shows a consistent group formation that is in accordance to the proposed functional classification. From direct comparison of both UPGMA distance trees we conclude that internal branches (branch lengths < 0.3 dits) are equally organized. Topological rearrangements are caused by swapped, longer branches. This might be caused by the generally tight distribution of dScores in both distance matrices obtained (0.666 ± 0.198 dits and 0.668 ± 0.197 dits for the 1bgj set and 1d7l set, respectively). Usually, energy profile alignments with biological significance (for example two proteins with a similar fold) yield a dScore of ≤ 2.5 dits. In comparison, the energy profile alignment shown in Fig. S2 corresponds to a dScore of 0.72. As shown in Equation 4 (Materials and Methods), slight variations of $\bar{x}_p$ can lead to slight variations of the dScore as well. To ensure reliable values of $\bar{x}_p$, dScore calculations have been carried out with 250 energy profile permutations. Since both distance matrices are highly similar (-0.002 ± 0.019 dits in dScore difference), the greedy tree generation equation used in UPGMA can be proposed as the most likely cause of topological rearrangements. Fig. S4 and Fig. S5 show that distance trees obtained from neighbor-joining clustering fit to the functional classification of PHBH with only few topological rearrangements.

To further investigate energy profile distance relationships, t-SNE (t-distributed stochastic neighbor embedding ((van der Maaten and van Hinton, 2008)) and classic multi-dimensional scaling (MDS) were employed for studying low-dimensional spatial embedding (configurations) of energy profile similarities (Fig. S6). Configurations were separately computed for each structure model set. Both t-SNE and MDS aim at finding a low-dimensional, human understandable configuration of the input data while preserving data similarities, and thus provide a readily understandable visualization of data relationships (energy profile similarities in this case). Although the objectives of these methods are the same, both techniques differ in the underlying embedding approach, giving a complementary view on the data. As depicted in Fig. S6, both embedding techniques found similar configurations for both structural model sets. Although no distinct non-overlapping energy profile clusters are visible, all profiles group according to the proposed coenzyme specificities. In comparison to NADPH-specific and NAD(P)H-dependent PHBHs, NADH-preferring PHBHs are found to be relatively dispersed in the embedding space, from which it

can be deduced that these enzymes yield larger functional variety. In all generated configurations, the largest inter-group distance is found between NADPH-specific and NADH-preferring PHBHs, leading to a consensus embedding with NADPH-specific and NADH-preferring PHBHs being separated by NAD(P)H-dependent PHBHs. This consensus embedding is in accordance with the general layout of generated distance trees. From this it can be proposed that NAD(P)H coenzyme specificity is an intermediate state between NADH and NADPH specificity.

2 Supplementary Figures

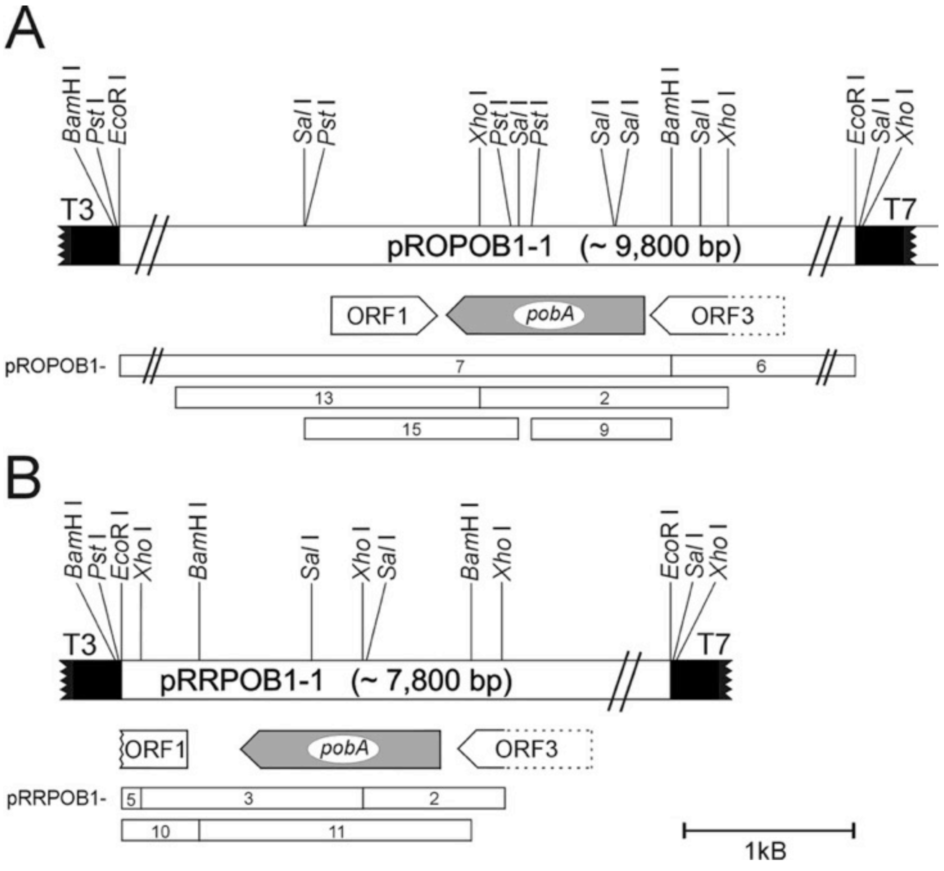

Figure S1 Overview of cloned PHBH encoding regions in *R. opacus* 557 (A) and *R. rhodnii* 135 (B). The *Eco*RI fragments cloned into pBluescript II SK (+) are shown as pROPOB1-1 (9.8 kb) or pRRPOB1-1 (7.8 kb) with some of the major restriction sites used for subcloning. Inserts of subclones are indicated by corresponding boxes.

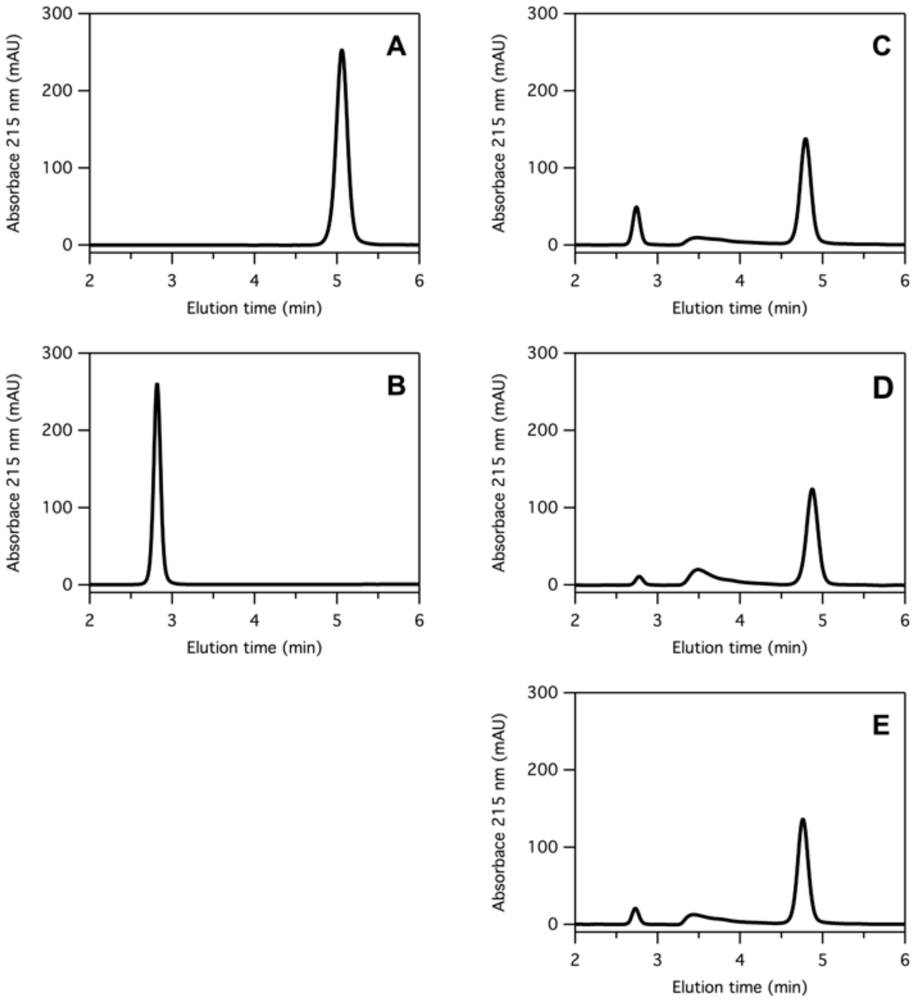

Figure S2 HPLC chromatograms from PHBH reactions. The substrate (4-hydroxybenzoate) (A) and product (3,4-dihydroxybenzoate) (B) were used as reference compounds. Enzymatic reactions with PHBH$_{Ro1CP}$ (C), PHBH$_{Cn1}$ (D) and PHBH$_{Cn2}$ (E) and 4-hydroxybenzoate as substrate. The elution times, as well as respective UV/Vis spectra, were used for identification. The HPLC chromatograms shown for the enzymatic reactions are obtained from samples taken after 5 min incubation time. Due to this limited time, only small amounts of product are formed. The tailing peak in-between product and substrate originates from the incubation buffer.

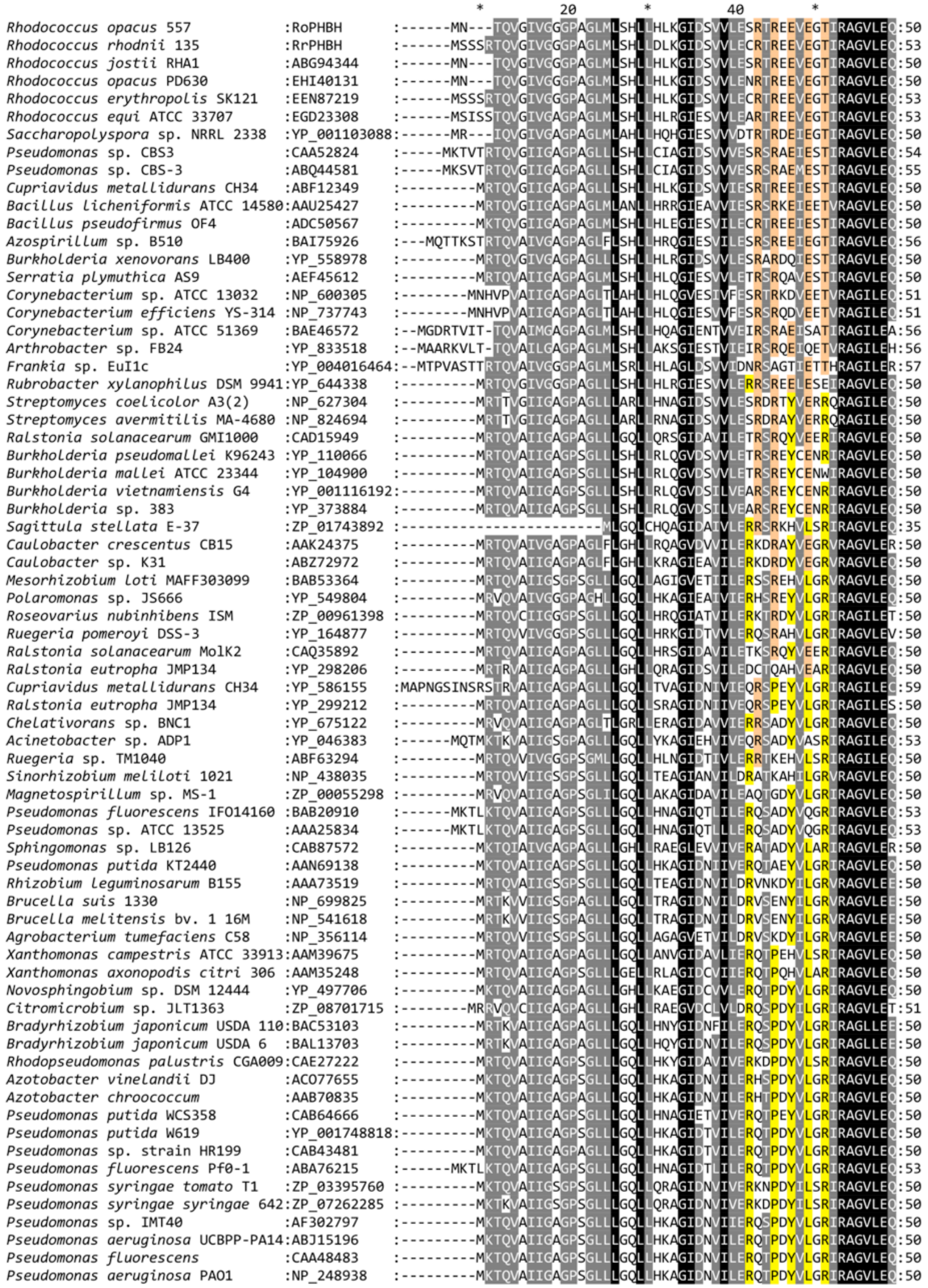

Figure S3a Multiple sequence alignment of the N-terminal part of selected PHBH amino acid sequences, which includes the fingerprint motifs for coenzyme recognition.

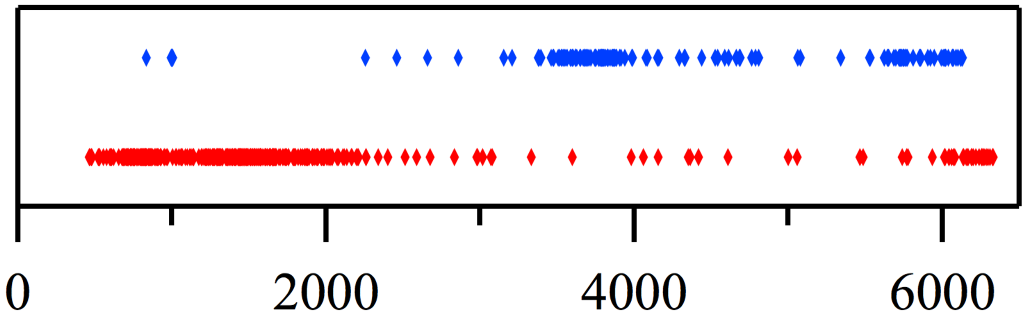

Figure S3b Distribution of PHBH sequences in the BlastP outputs using $PHBH_{Pf}$ or $PHBH_{Ro}$ as query sequence, respectively. Blue markers, distribution of sequences found in the *Pf*-dataset, but not in the *Ro*-dataset (145 sequences out of 6135 sequences). Red markers, distribution of sequences found in the *Ro*-dataset, but not in the *Pf*-dataset (347 sequences out of 6337 sequences).

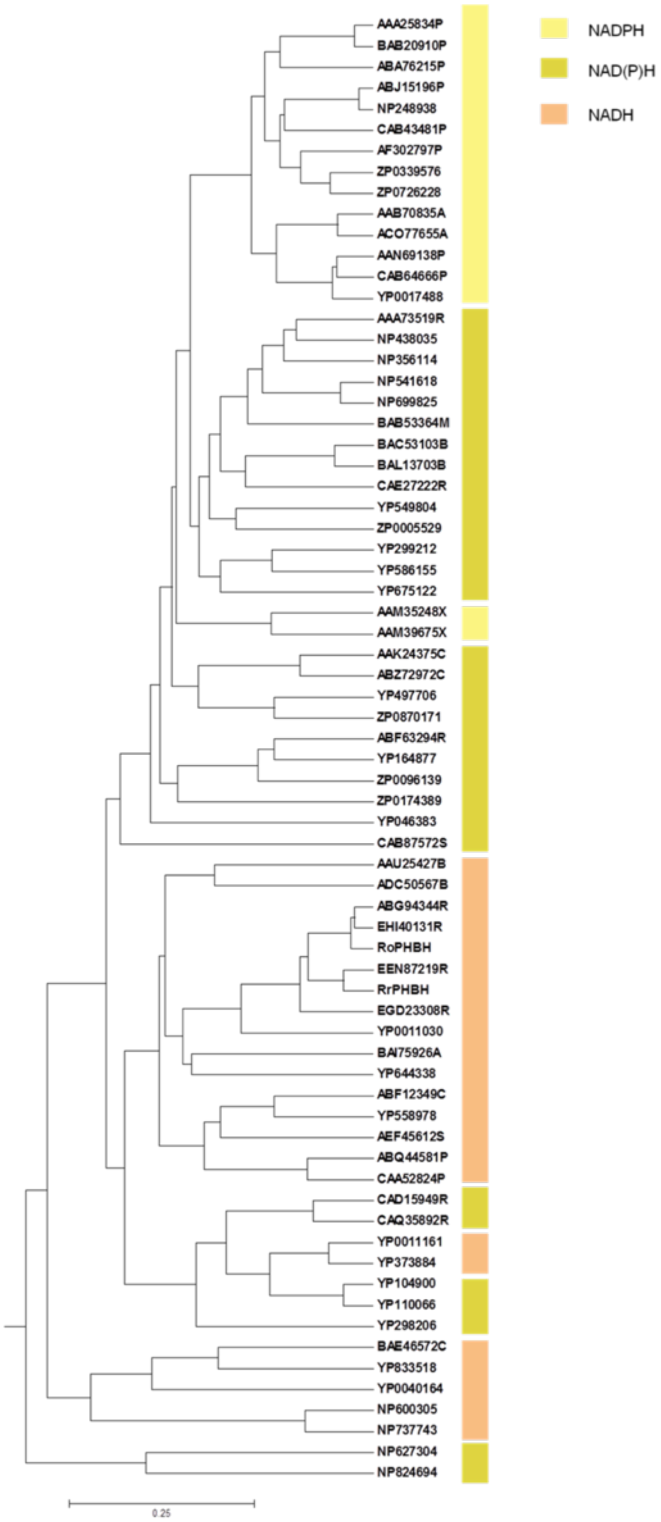

Figure S4 Distance tree of energy profiles generated using UPGMA hierarchical clustering. Protein energy profiles were calculated from PHBH model structures obtained by homology modeling with a known PHBH structure (pdb: 1d7l) serving as modelling template. dScores were applied as a measure of energy profile distance. Coloring is in accordance to functional classification.

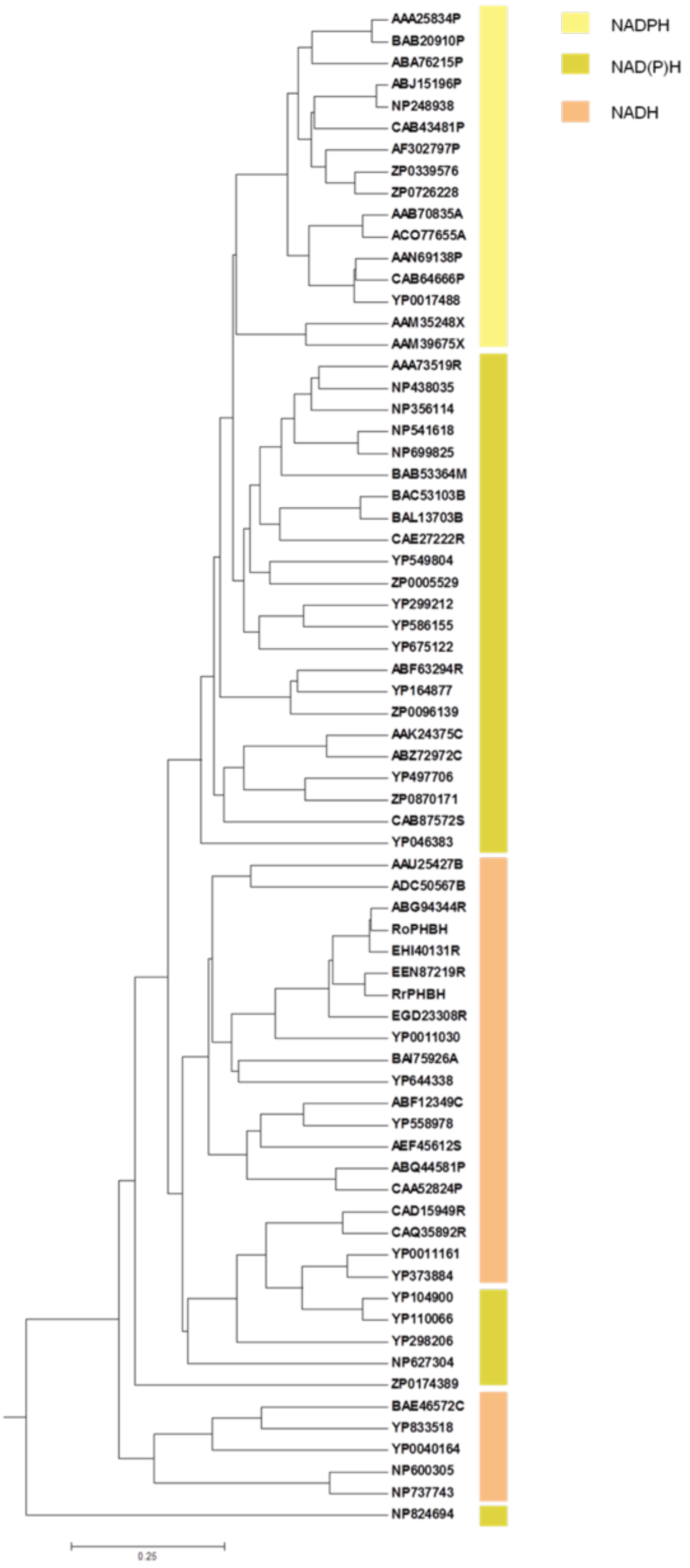

Figure S5 Distance tree of energy profiles generated using UPGMA hierarchical clustering. Protein energy profiles were calculated from PHBH model structures obtained by homology modeling with a known PHBH structure (pdb: 1bgj) serving as modelling template. dScores were applied as a measure of energy profile distance. Coloring is in accordance to functional classification.

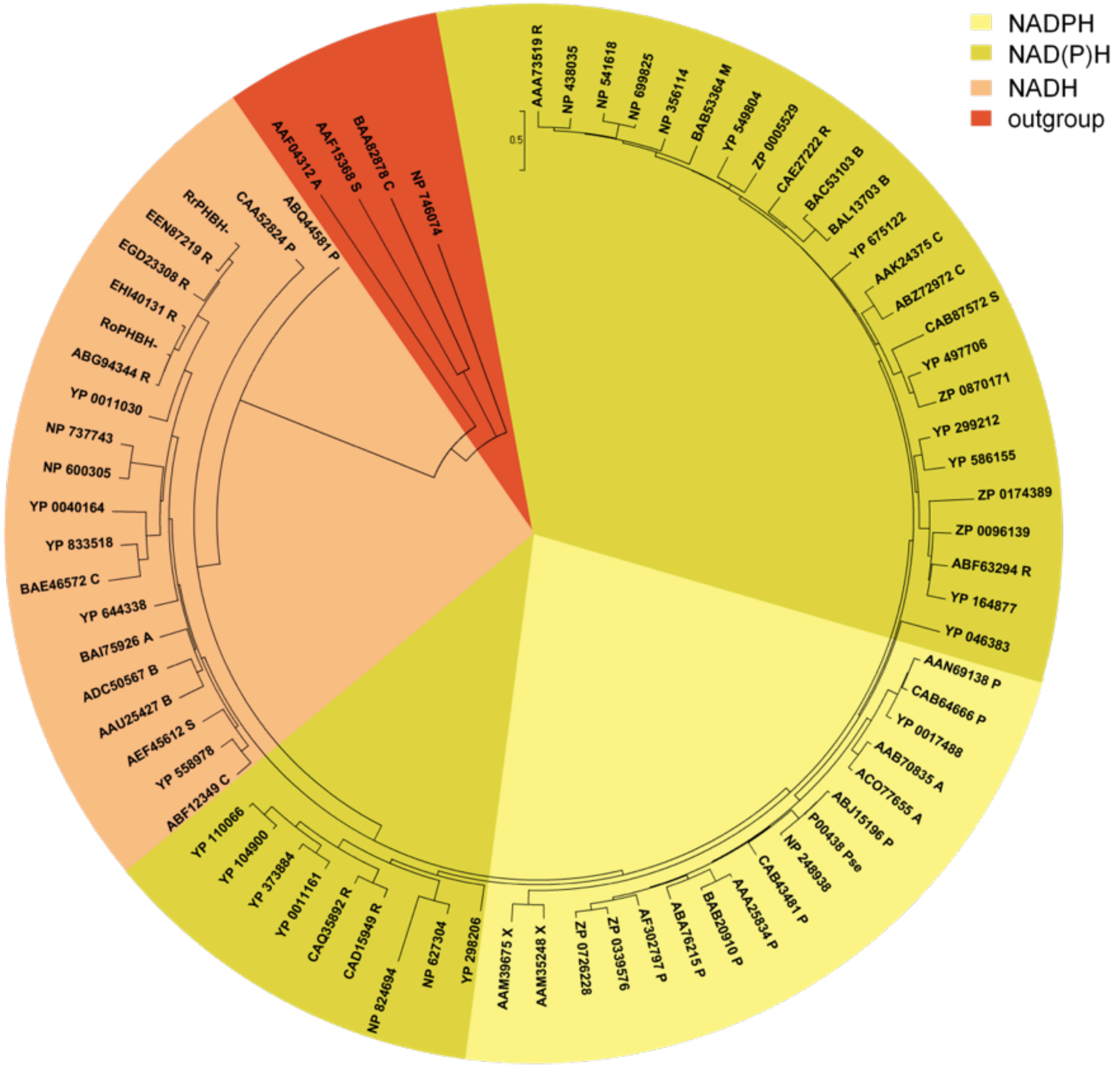

Figure S6 Distance tree of energy profiles generated using Neighbor joining hierarchical clustering. Protein energy profiles were calculated from PHBH model structures obtained by homology modeling with a known PHBH structure (pdb: 1d7l) serving as modelling template. dScores were applied as a measure of energy profile distance. Coloring is in accordance to functional classification. Energy profiles of outgroup proteins have been predicted from sequence (Heinke and Labudde, 2013) since no model structures could be generated due to low sequence identities to the modelling template.

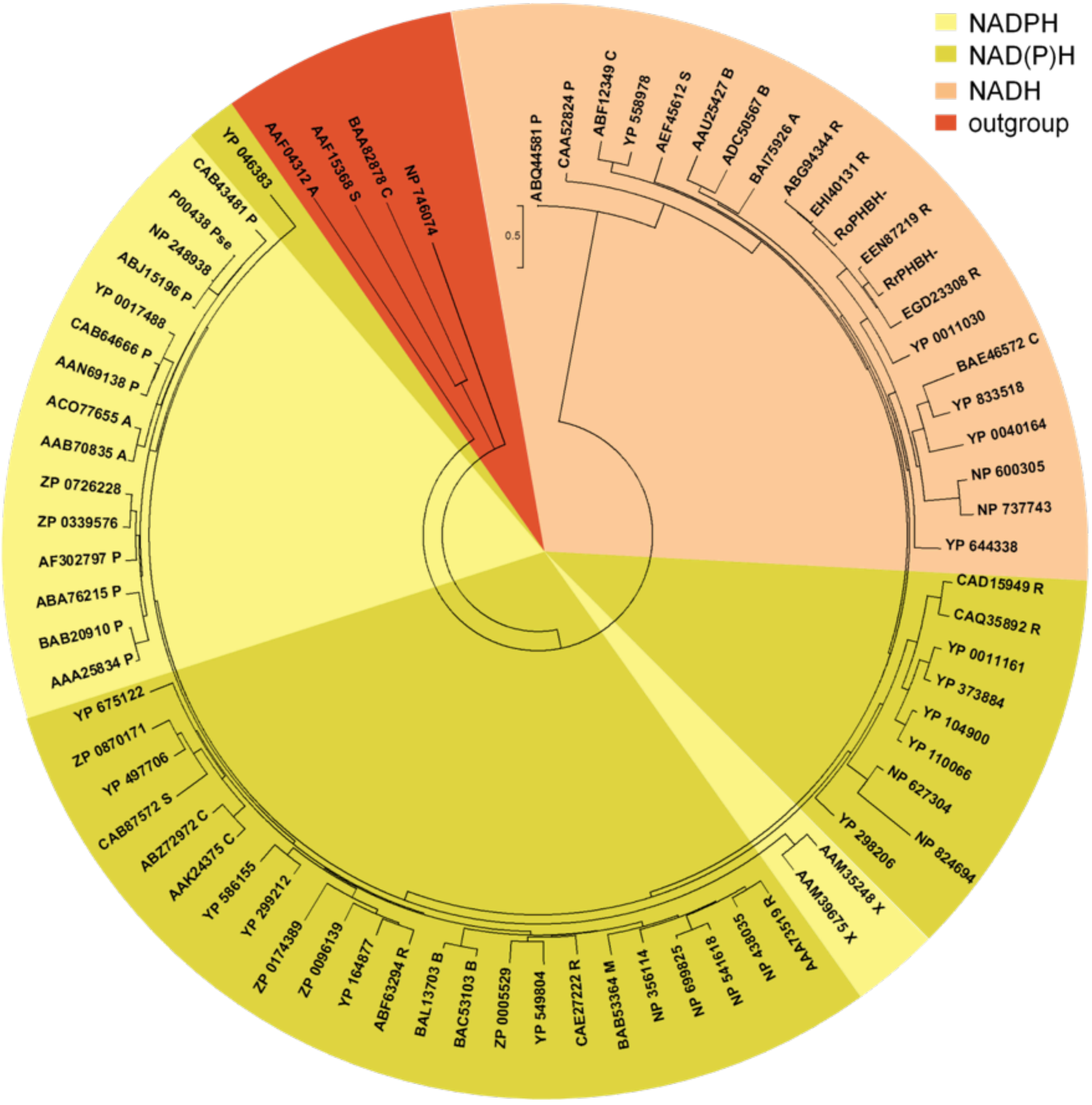

Figure S7 Distance tree of energy profiles generated using Neighbor joining hierarchical clustering. Protein energy profiles were calculated from PHBH model structures obtained by homology modeling with a known PHBH structure (pdb: 1bgj) serving as modelling template. dScores were applied as a measure of energy profile distance. Coloring is in accordance to functional classification. Energy profiles of outgroup proteins have been predicted from sequence (Heinke and Labudde, 2013), since no model structures could be generated due to low sequence identities to the modelling template.

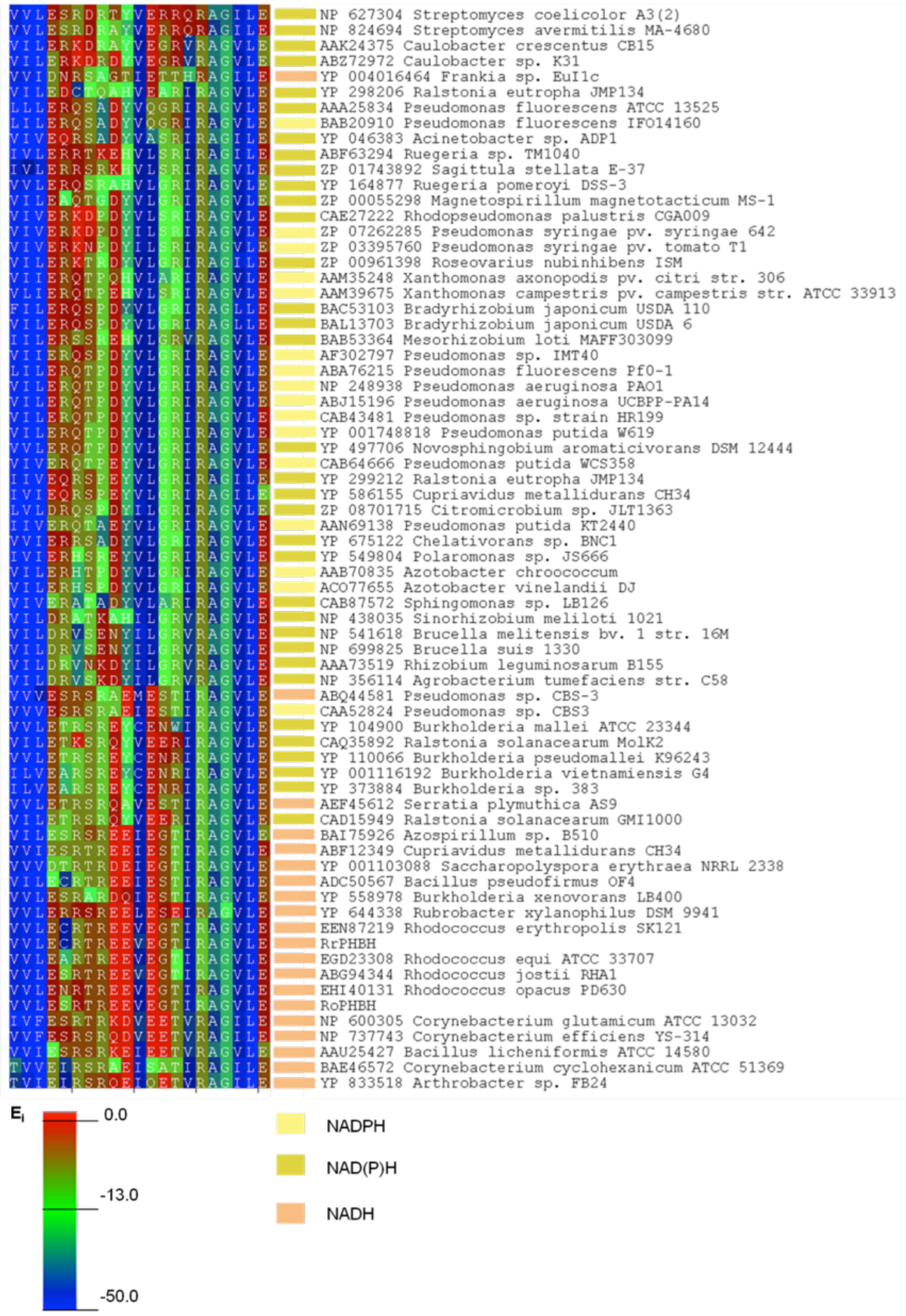

Figure S8 Multiple energy profile alignment of the PHBH fingerprint motif. Energy profile data has been derived from the 1bgj structure model set.

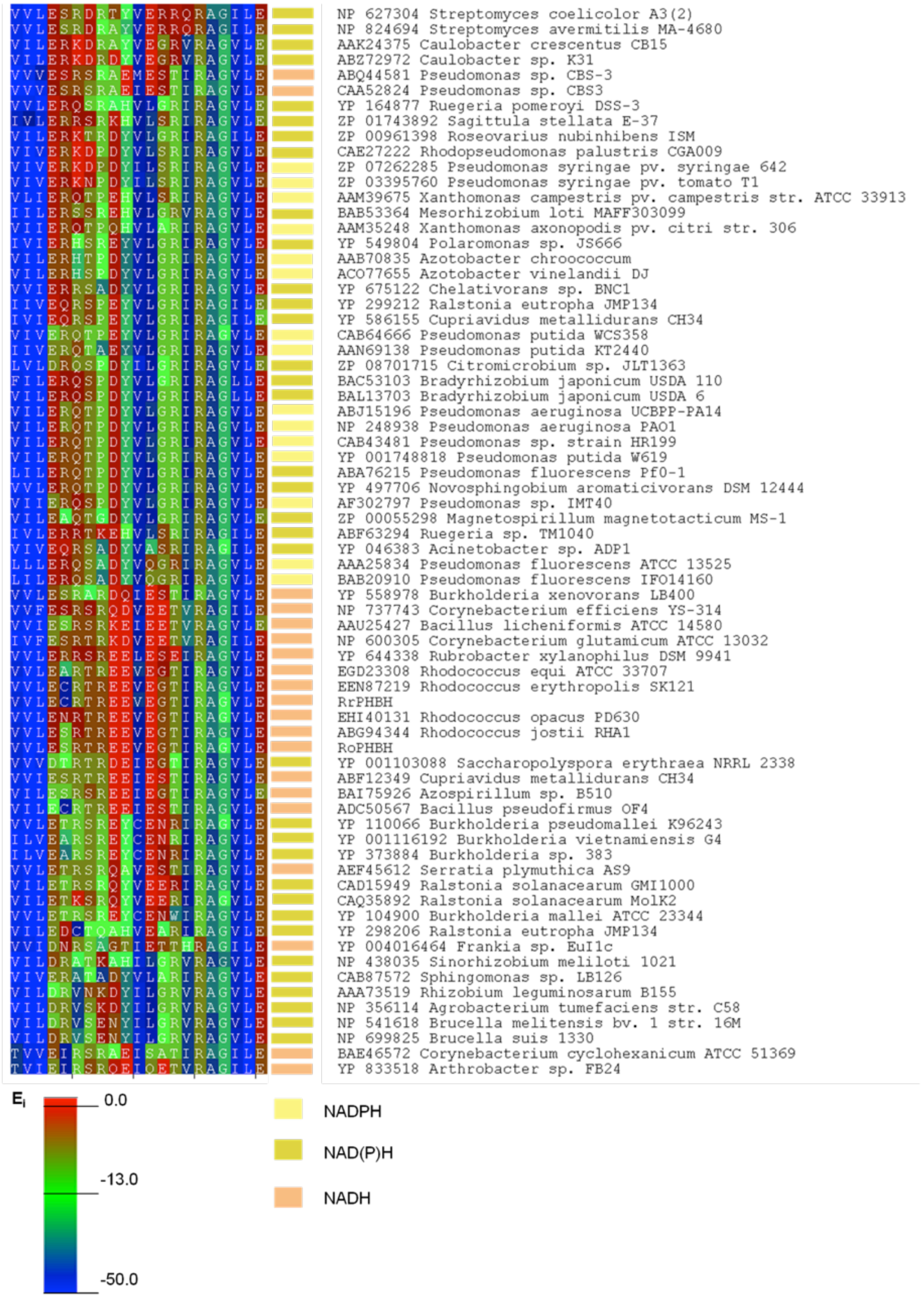

Figure S9 Multiple energy profile alignment of the PHBH fingerprint motif. Energy profile data has been derived from the 1d7l structure model set.

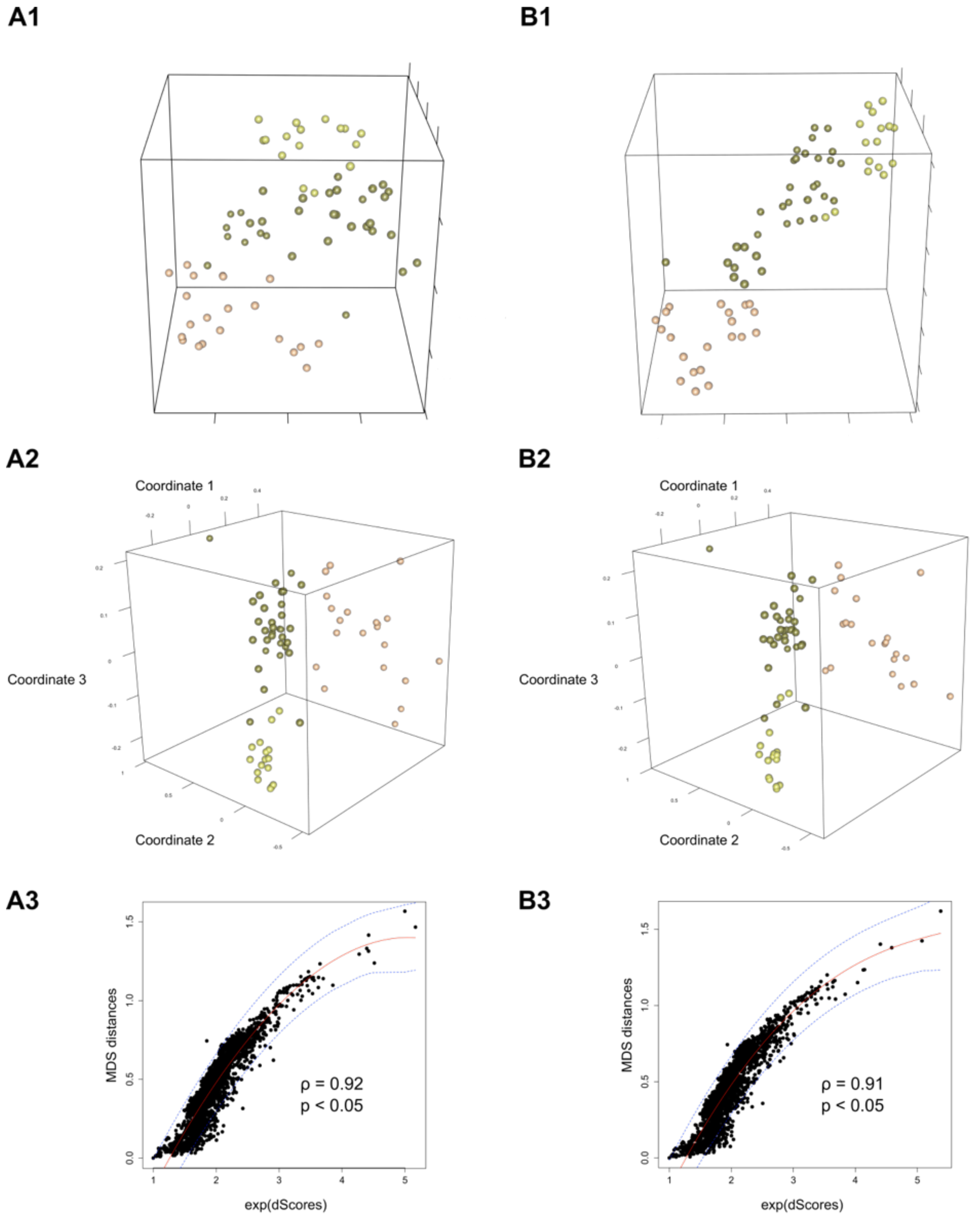

Figure S10 Visualization of energy profile similarities using three-dimensional embedding. Embeddings have been individually computed for each model structure set (A: 1bgj model structure set, B: 1d7l model structure set). t-SNE (van der Maaten and van Hinton, 2008) (plots A1 and B1) and classic multi-dimensional scaling (MDS, plots A2 and B2) have been employed as embedding techniques. Pairwise dScores have been used as input. Each point in the three-dimensional plots

corresponds to an energy profile in the dataset. The plots A3 and B3 show the quality of the MDS embedding by means of evaluating the correlation of input dScores and resulting inter-point distances (which would result to a perfect correlation fit if MDS finds an error-free configuration). For each dataset, MDS finds meaningful configurations as indicated by Spearman's rank correlation coefficients. P-values have been determined by permutation testing. However, correlation analyses of t-SNE configurations is of no use, because t-SNE computes low-dimensional configurations by means of minimizing inter-point information differences, rather than minimizing inter-point distance errors, as in case of MDS. t-SNE parameters have been optimized by grid search over the parameter space with respect to the resulting embedding error.

All configurations show well-structured groupings of energy profiles corresponding to co-enzyme specificities (color highlighting as in Fig. S8 and Fig. S9), which, on the one hand, support the distance trees depicted in Fig. S4 – Fig. S7, and, on the other hand, support experimental data.

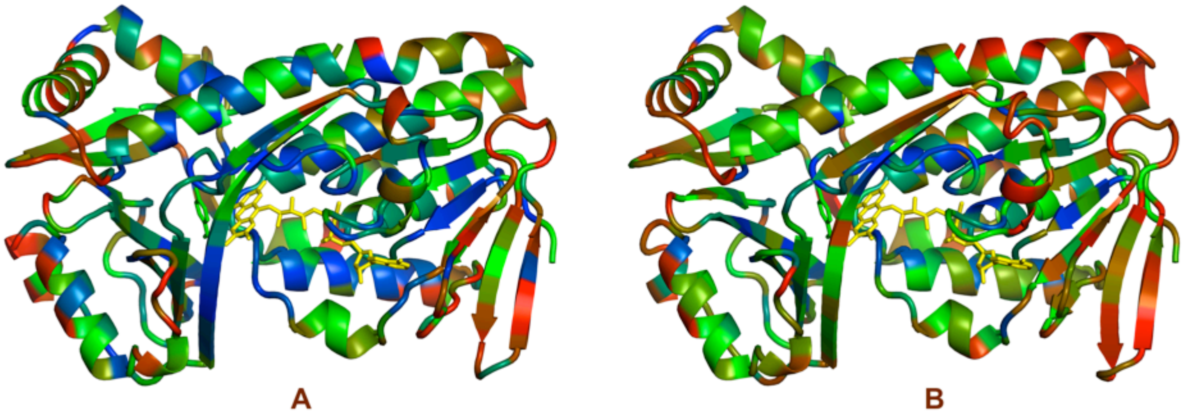

Figure S11 Cartoon images of (A), the crystal structure of PHBH$_{Pf}$ and (B), a structural model of PHBH$_{Ro}$, colored according to the evolutionary rates calculated with the Rate4site program. The color ranges from blue, highly conserved residues, via green to red, highly variable residues. The bound substrate is colored green and the FAD cofactor yellow.

References

Heinke, F., and Labudde, D. (2013). "Functional analyses of membrane protein mutants involved in nephrogenic diabetes insipidus: An energy-based approach", in: *Research on Diabetes.* iConcept Press, Hong Kong).

van der Maaten, L.J.P., and van Hinton, G.E. (2008). Visualizing high-dimensional data using t-SNE. *J. Mach. Learn. Res.* 9**,** 2579-2605.
